# The therapeutic potential of transposable element activity modulation

**DOI:** 10.1038/s44321-026-00528-z

**Published:** 2026-10-03

**Authors:** Gabriele Morelli, Marco D’Onghia, Arianna Mangiavacchi, Valerio Orlando

**Affiliations:** King Abdullah University of Science and Technology (KAUST), Biomedical Science (BioMed) Division, Thuwal, 23500-6900 Kingdom of Saudi Arabia

**Keywords:** Immunology

## Abstract

The reactivation of transposable elements (TEs) is a recurrent molecular event across cancer, neurodegeneration, autoimmune disease, and aging, yet its therapeutic implications remain largely unexplored. Normally repressed under strict epigenetic surveillance, reactivated transposable elements can generate immunostimulatory nucleic acids that engage cytosolic pattern-recognition receptors, converging on type I interferon signaling and driving chronic sterile inflammation. Yet this inflammatory potential is inherently dual: the same reactivation that drives pathology in some contexts can be harnessed to prime immunity in others. Here, we review two complementary strategies that exploit this duality. Suppressive approaches, including nucleoside reverse transcriptase inhibitors, antisense oligonucleotides, and epigenetic silencing agents, aim to quench pathological TE-driven inflammation in diseases and restore other adaptive mechanisms governing homeostasis. Conversely, controlled TE reactivation through epigenetic priming can induce a viral mimicry state in tumor cells, sensitizing them to immune checkpoint blockade. In parallel, TE-derived peptides presented on MHC class I molecules are emerging as a largely unexploited reservoir of shared tumor antigens for next-generation vaccines and engineered T cell therapies. Together, these observations reframe transposable elements not as mere viral relics to be tolerated, but as a tunable immunological switch whose therapeutic modulation holds promise across some of the most challenging diseases of our time.

## Abstract (web summary)

In this Review, V. Orlando and colleagues discuss how transposable elements function as tunable regulators of immunity and explore therapeutic strategies that either suppress or reactivate their activity to treat diverse human diseases.

## Graphical abstract

**Figure:**
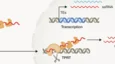

## Introduction

**Figure 1:**
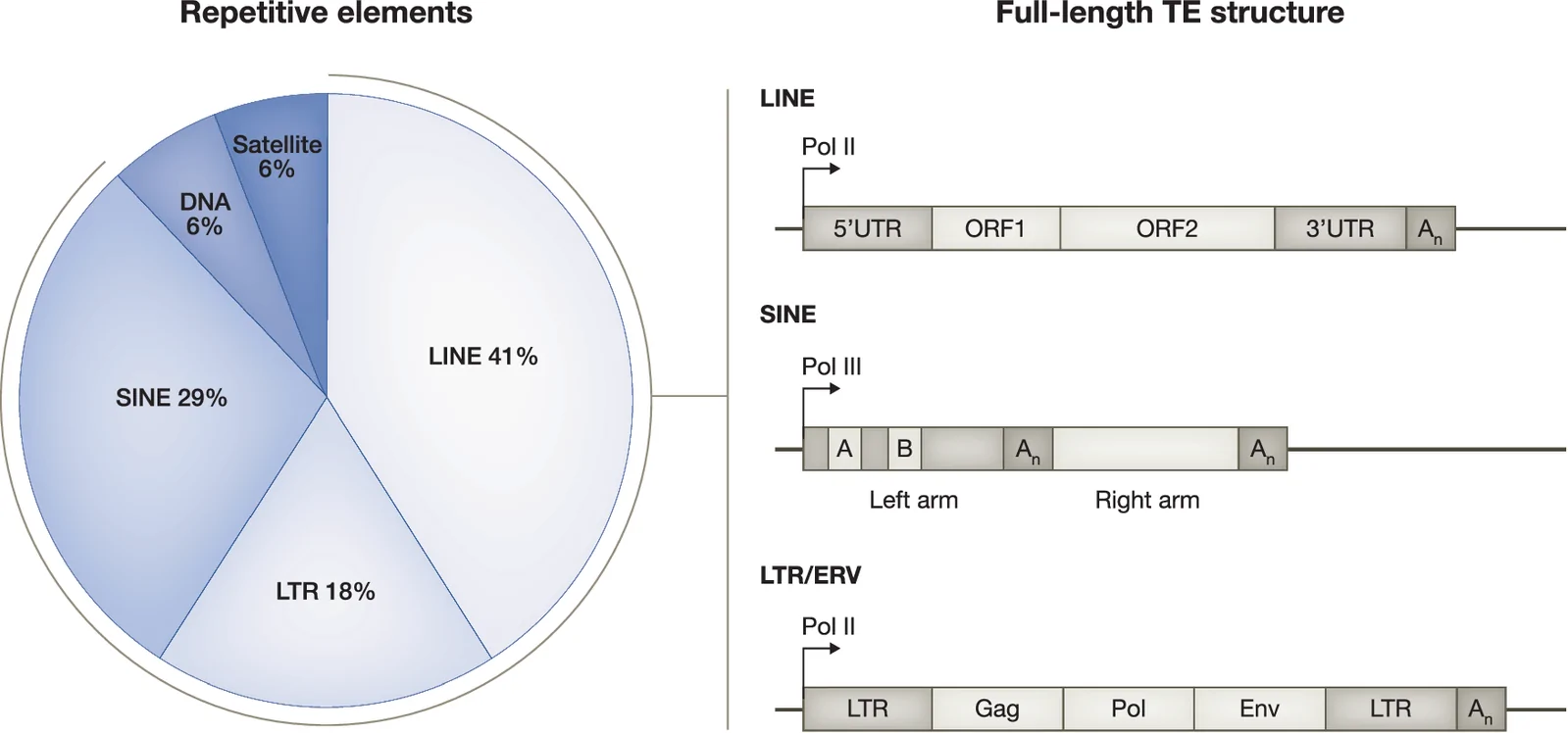
Relative abundance and canonical full-length structure of human retrotransposons. (Left) Transposable elements are the largest component of the repetitive fraction of the human genome. Retrotransposons predominate, comprising long interspersed nuclear elements (LINEs, ~41%), short interspersed nuclear elements (SINEs, ~29%) and long terminal repeat (LTR) retroelements including endogenous retroviruses (~18%), while DNA transposons (~6%) and satellite repeats (~6%) account for the remainder. (Right) Structural organization of full-length elements from the three principal retrotransposon classes. LINEs are transcribed by RNA polymerase II and contain a 5′ untranslated region (UTR) with internal promoter activity, two open reading frames (ORF1 and ORF2, the latter encoding the endonuclease and reverse transcriptase activities required for autonomous retrotransposition), a 3′UTR and a poly(A) tail (A_n_). SINEs are transcribed by RNA polymerase III from internal A- and B-box promoter elements and are non-autonomous, depending on the LINE-1 machinery for mobilization; the composite structure comprises a left and a right arm, each terminating in an A_n_ stretch. LTR/ERV elements are transcribed by RNA polymerase II and are flanked by two LTRs that harbor promoter and enhancer activity, with internal *gag*, *pol*, and *env* genes characteristic of their retroviral ancestry.

A substantial fraction of the human genome remains only partially understood, forming an increasingly vast regulatory landscape largely dominated by transposable elements (TEs) (De Koning et al, 2011). TEs are mobile genetic elements, classified according to their mobilization mechanism: DNA transposons replicate through a direct excision and reintegration mechanism (“cut-and-paste”), whereas retrotransposons amplify via an RNA intermediate that is reverse transcribed and inserted at new genomic sites (“copy-and-paste”). The latter group includes long terminal repeat (LTR) elements and non-LTR elements, such as long interspersed nuclear elements (LINEs) and short interspersed nuclear elements (SINEs) (Fig. 1). Among retrotransposons, LINE-1 elements, which constitute ~20% of the human genome, represent the only class in humans that retains autonomous retrotransposition activity (Hancks and Kazazian, 2012). These elements encode the proteins required for their own mobilization and follow a multistep life cycle involving transcription, cytoplasmic translation, and nuclear reintegration through target-primed reverse transcription. In contrast, SINEs lack coding capacity and rely on LINE-encoded proteins for their propagation (Kramerov and Vassetzky, 2011). LTR elements, including endogenous retroviruses, share structural similarities with exogenous retroviruses, although in humans, they are inactive in terms of retrotransposition (Magiorkinis et al, 2015). Among LTRs, human endogenous retroviruses (HERVs), ~8% of the human genome, are the best-characterized in the context of human disease (Dopkins and Nixon, 2024).

Despite their initial identification by the Nobel Laureate Barbara McClintock (McClintock, 1950) as a major source of genetic variation in evolution, TEs were long regarded as inert, potentially dangerous genome components, remnants of ancient viral genomes. Only recently, thanks to a broader, multidimensional approach to genome structure and function, supported by major advances in sequencing and bioinformatic resources, TEs have been gaining increasing attention. Indeed, in contrast to initial beliefs, TE activity and, in particular, their RNAs have been reported to be expressed in somatic cells (Faulkner et al, 2009; Muotri et al, 2005; Coufal et al, 2009), being integral parts of core cellular functions both in early and adult life. Different classes of TE-derived RNAs have been shown to be part of fundamental mechanisms of cell identity programs and control of tissue homeostasis by exerting enhancer functions, regulating chromatin remodeling enzymes and modulating the innate immune response (Grow et al, 2015; Jachowicz et al, 2017; Sundaram et al, 2017; Diehl et al, 2020; Qiu and Köhler, 2020; Gebrie, 2023; Lawson et al, 2023; Fueyo et al, 2025; Feschotte, 2026). Recently, owing to their emerging roles in stress-induced gene expression plasticity and the regulation of inflammatory responses, TEs have increasingly been recognized as bona fide components of cellular resilience mechanisms, an aspect of growing relevance to aging and age-related pathologies (Gorbunova et al, 2021; Della Valle et al, 2025).

TE activity is normally constrained by multiple layers of epigenetic regulation, including DNA methylation, repressive histone modifications, and sequence-specific recognition by KRAB zinc-finger proteins, which recruit co-repressor complexes to enforce heterochromatin formation (Slotkin and Martienssen, 2007; Guelen et al, 2008; Rowe et al, 2013; Imbeault et al, 2017). Central to this repressive cascade is SETDB1, the H3K9 methyltransferase recruited by TRIM28/KAP1 that deposits the H3K9me3 mark at TE loci to enforce heterochromatin formation.

Additional layers of control include RNA-mediated pathways such as PIWI-interacting RNAs (piRNAs) in the germline and the HUSH complex, which targets actively transcribed elements (Ozata et al, 2019; Müller and Helin, 2024). Because of their documented importance in both developmental and adaptive mechanisms, transposable elements are subject not only to this tight negative regulation but also to equally complex activation pathways (Li et al, 2024).

**Figure 2:**
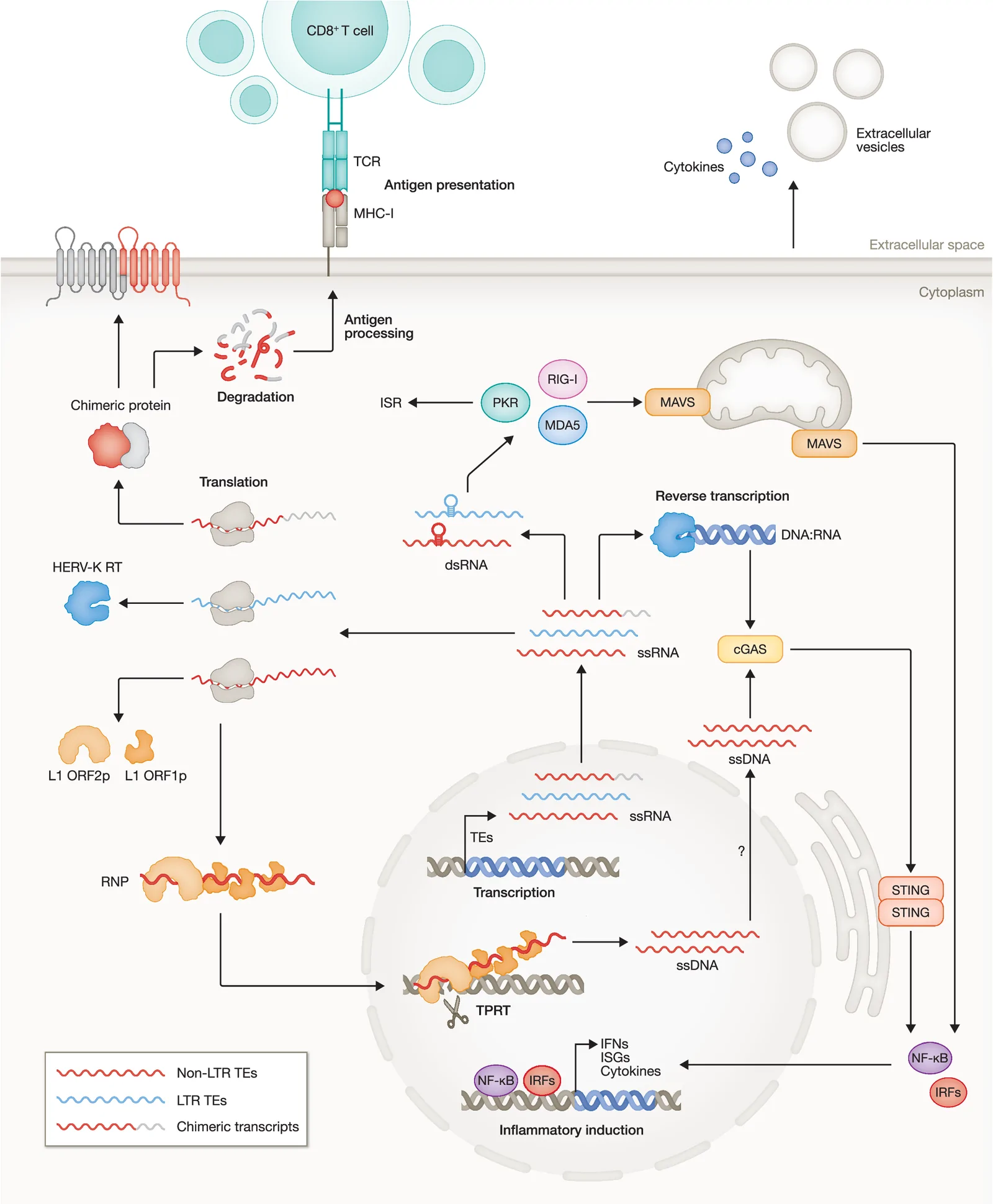
Mechanisms by which transposable element reactivation triggers innate immune and inflammatory responses. Derepressed TEs generate immunostimulatory molecules through multiple routes. TE transcription produces single-stranded RNAs (ssRNA) that can form double-stranded RNA (dsRNA) structures or serve as templates for reverse transcription, generating cytoplasmic DNA:RNA hybrids. Moreover, single-stranded DNA (ssDNA) molecules, generated by target-primed reverse transcription (TPRT), can accumulate in the cytoplasm, although the precise mechanisms remain to be elucidated. dsRNA is sensed by RIG-I and MDA5, which signal through the mitochondrial antiviral signaling (MAVS) protein, or by PKR, which engages the integrated stress response (ISR). Cytoplasmic DNA species are sensed by cGAS, which signals through STING. These pathways converge on NF-κB and interferon regulatory factor (IRF) activation, driving the expression and subsequent secretion of interferons, interferon-stimulated genes (ISGs), and pro-inflammatory cytokines. In parallel, translation of TE-gene chimeric transcripts can generate peptides that are processed and presented on MHC class I molecules, expanding the repertoire of antigens recognized by CD8⁺ T cells. Created with BioRender.com.

Disruption of negative control systems leads to TE reactivation, which can result in insertional mutagenesis, chromosomal rearrangements, and aberrant gene regulation (Beck et al, 2011; Kassiotis, 2014). TE dysregulation has been consistently observed across a wide spectrum of pathological conditions, including cancer (Burns, 2017), autoimmune and metabolic disorders (Mustelin and Ukadike, 2020; Chen et al, 2025; Daouk et al, 2025), neurodegeneration (Jönsson et al, 2020), fibrosis (Rostami and Bradic, 2021), and aging-related dysfunction (Gorbunova et al, 2021; Della Valle et al, 2022), all of which share inflammation as a common feature. The chronic innate immune activation observed in these contexts probably reflects the combined failure of multiple RNA surveillance mechanisms, with TE derepression representing one important contributor. Indeed, mammalian cells continuously eliminate a wide range of aberrant endogenous transcripts, including TE-derived RNAs as well as cryptic, misprocessed, or intergenic transcripts, through multiple quality control mechanisms that operate upstream of innate immune sensing (Radrizzani et al, 2024). When these surveillance pathways fail, these “unwanted” nucleic acids accumulate in the cytoplasm and trigger an inflammatory response. TE-derived transcripts are particularly prone to escaping surveillance due to their AU-rich, intronless character and capacity to form dsRNA structures or generate cytoplasmic cDNA intermediates; however, they are unlikely to be the sole drivers of this response. Mechanistically, TE transcripts may contribute to inflammatory responses by either generating double-stranded RNA (dsRNA) molecules, or by reverse transcription, producing DNA intermediates that accumulate outside the nucleus (Zhao et al, 2018; De Cecco et al, 2019; Volkmann et al, 2020; Gazquez-Gutierrez et al, 2021). These molecules resemble viral genomes and are thus sensed by pattern-recognition receptors, engaging distinct sensing pathways: LINE-1 cDNA, produced by reverse transcription of LINE-1 RNA intermediates and recently shown to arise from noncanonical ORF2p activity in the cytoplasm (Baldwin et al, 2024), is the proximate ligand for the DNA sensor cGAS, whereas dsRNA structures present within TE transcripts activate the cytosolic RNA sensors MDA5, RIG-I and PKR (Gazquez-Gutierrez et al, 2021). Both pathways converge on a shared type I interferon (IFN-I) response, making their combined output the principal immunostimulatory consequence of TE derepression (Fig. 2).

Beyond direct sensing, TEs can influence immune responses by acting as regulatory elements that drive IFN-stimulated gene expression and by generating peptides presented on major histocompatibility complex class I (MHC-I) molecules (Larouche et al, 2024; Horton et al, 2023). Notably, TE-derived peptides presented on MHC-I can expand the repertoire of tumor-associated antigens available for immune recognition, a concept that will be discussed further in the context of cancer-targeted therapy. This intrinsic immunogenicity places TEs in a unique position at the boundary between self and non-self and suggests that this property may have been co-opted to provide cells with a tunable source of “quasi-non-self” signals, whose controlled activation could provide a rapid and cell-autonomous means to initiate inflammatory responses when needed. When transient and controlled, TE-induced inflammation may contribute to physiological signaling states, such as those observed in tissue repair (Mangiavacchi et al, 2024). However, when TE regulation is compromised, sustained production of immunogenic nucleic acids can lead to persistent activation of innate immune pathways and chronic, sterile inflammation. Altogether, the spatially and temporally regulated transcriptional activity of TEs appears to influence vital aspects of cell and tissue physiology common to a broad spectrum of high-impact pathologies and to aging, making TEs potential new targets for therapeutic purposes.

In this review, we discuss how these converging observations have prompted growing interest in the therapeutic modulation of TE activity. We explore how both suppression and controlled reactivation of TEs may be leveraged to preserve or restore cell identity programs and regulate inflammatory responses contributing to tissue homeostasis and, ultimately, healthspan.

## Therapeutic suppression of transposable elements across human diseases

Therapeutic strategies aimed at limiting TE activity primarily focus on reducing the production or persistence of TE-derived nucleic acids. These approaches can be broadly categorized into three complementary modalities. First, sequence-specific targeting of TE transcripts can be achieved using antisense oligonucleotides (ASOs) or small interfering RNAs (siRNAs), which promote RNA degradation or prevent translation. Second, pharmacological inhibition of reverse transcription, most notably through nucleoside reverse transcriptase inhibitors (NRTIs) originally developed for the treatment of HIV infection, can limit the generation of TE-derived DNA intermediates and their downstream effects. Third, modulation of the epigenetic landscape using small molecules can reinforce transcriptional silencing of TEs by restoring repressive chromatin states. Collectively, these approaches provide a versatile framework to reduce TE activity, whose therapeutic potential is now being explored across a wide spectrum of pathological conditions characterized by TE dysregulation.

**Figure 3:**
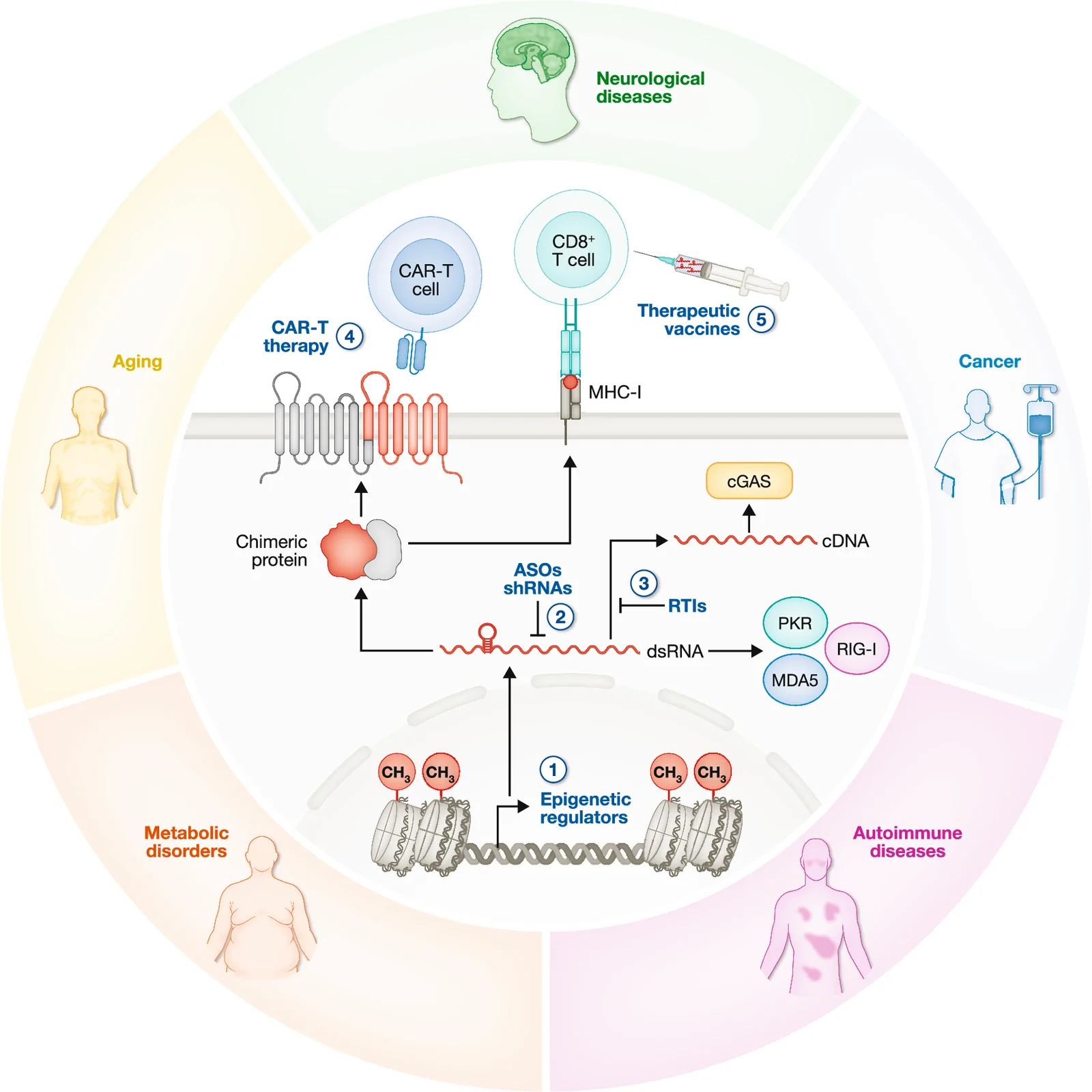
Potential TE-targeting therapeutic tools across different disease contexts. Five therapeutic strategies targeting the transposable element activity axis are illustrated across five disease contexts: cancer, neurological diseases, autoimmune diseases, metabolic disorders, and aging. (1) Epigenetic modulators act at the chromatin level to either reinforce TE silencing, by restoring repressive DNA methylation and histone modifications, or to reactivate TEs in a controlled manner to enhance tumor immunogenicity. (2) Antisense oligonucleotides (ASOs) and short hairpin RNAs (shRNAs) target TE-derived transcripts for sequence-specific degradation, preventing the accumulation of immunostimulatory ssRNA and dsRNA species and the downstream production of chimeric proteins. (3) Reverse transcriptase inhibitors (RTIs) block the conversion of TE RNA intermediates into cytoplasmic cDNA, thereby preventing activation of the cGAS-STING DNA sensing pathway. (4) CAR-T cell therapy exploits TE-derived chimeric proteins presented on MHC class I molecules as tumor-specific antigens, directing engineered cytotoxic T cells against antigen-expressing cells. (5) Therapeutic vaccines prime CD8⁺ T cell responses against TE-derived peptides displayed on MHC-I, harnessing the immunogenicity of shared TE-encoded antigens to mount a targeted adaptive immune response. Created with BioRender.com.

A key consideration, however, is that a substantial fraction of TE-derived sequences has been co-opted during evolution to function as enhancers, promoters, and other regulatory elements that are integral to normal gene expression programs (Chuong et al, 2016). Consequently, broad suppression of TE activity could inadvertently disrupt these physiological functions, highlighting the need for strategies capable of selectively targeting pathologically reactivated elements while sparing exapted regulatory sequences. A second important consideration should be made at the outset: in most disease contexts discussed in this review, with the notable exception of cancer, the pathogenic evidence concerns TE transcriptional activity (the production of TE-derived RNAs, dsRNAs, and cytoplasmic cDNA intermediates that engage innate immune sensors) rather than insertional mutagenic retrotransposition. These two phenomena are mechanistically distinct, and therapeutic strategies targeting one do not necessarily address the other. An overview of these therapeutic modalities across the disease contexts discussed is provided in Fig. 3, while interventional and observational studies evaluating TE-targeting strategies across these diseases are summarized in Table 1.

**Table 1:** Interventional and observational studies evaluating transposable element-targeting therapeutic strategies across human diseases.

Trial/Reference	Condition	Therapeutic strategy	Phase	Main Outcome
**Cancer**				
NCT00964002 (Houédé et al, 2014)	Metastatic castration-resistant prostate cancer	Efavirenz (NNRTI)	Phase II	Subset of patients showed PSA stabilization
NCT03144804 (Parikh et al, 2020)	TP53-mutant metastatic colorectal cancer	Lamivudine (NRTI)	Phase II	Disease stabilization in a fraction of patients
NCT01878890	Solid tumors and non-Hodgkin lymphoma	Efavirenz (NNRTI) - dose escalation	Phase I	Tolerability established up to 1800 mg; pharmacological basis for subsequent studies
ACTRN12625001259437 (Lombard et al, 2025)	High-grade serous ovarian cancer	Efavirenz (NNRTI)	Phase II (ongoing)	Ongoing; results pending
NCT02007200 (Rozek et al, 2019)	Head and neck squamous cell carcinoma	Soy isoflavone supplementation	Phase II	Increased LINE-1 methylation; potential chemopreventive effect
**Neurological diseases**				
NCT04552795 (Sullivan et al, 2025)	Early Alzheimer’s disease	Lamivudine (NRTI)	Phase IIa pilot	Well tolerated; reductions in neuroinflammatory markers; changes in amyloid-related biomarkers
NCT06519357	Alzheimer’s disease	Lamivudine (NRTI)	Phase II (ongoing)	Ongoing; endpoints: neurocognitive biomarkers and IFN-stimulated gene expression
Chow et al, 2024	Alzheimer’s disease	NRTIs	Retrospective cohort study	NRTI exposure associated with lower AD incidence in HIV patients vs. controls; effect not seen with other antiretroviral classes
Magagnoli et al, 2025	Alzheimer’s disease	NRTIs	Retrospective cohort study	Each additional year of NRTI exposure → 4-13% reduction in AD risk; effect specific to NRTIs
NCT02868580 (Garcia-Montojo et al, 2021)	Amyotrophic lateral sclerosis	Abacavir + lamivudine + dolutegravir (Triumeq, NRTIs)	Phase II	Significant reduction in HERV-K expression; trend toward slower progression in virological responders
NCT05193994 (Lighthouse II)	Amyotrophic lateral sclerosis	Abacavir + lamivudine + dolutegravir (Triumeq, NRTIs)	Phase III (terminated early)	No benefit on survival at interim analysis (April 2025); trial terminated
**Autoimmune diseases**				
NCT06356740	Systemic lupus erythematosus	Abacavir + lamivudine (NRTIs) added to standard of care	Phase II (ongoing)	Ongoing; primary endpoint: reduction of IFN-I signature over 6 months
NCT02363452 (Rice et al, 2018)	Aicardi-Goutières syndrome	Abacavir + lamivudine + zidovudine (NRTIs)	Phase II	Transient reduction in IFN-I signature; effect rebounded after treatment cessation
NCT04731103 (Crow et al, 2025)	Aicardi-Goutières syndrome	NRTI monotherapy or short-term combination	Phase II	No sustained reduction in IFN-stimulated gene expression; limited by poor compliance
NCT02782858 (Hartung et al, 2022)	Multiple sclerosis	Temelimab (anti-HERV-W Env monoclonal antibody)	Phase II	No reduction in acute inflammation; higher doses: fewer T1-hypointense lesions and trends toward reduced brain atrophy
**Metabolic diseases**				
Ambati et al, 2020	Type 2 diabetes	NRTIs	Meta-analysis	NRTI exposure associated with significantly reduced incidence of diabetes
Magagnoli et al, 2023	Type 2 diabetes	NRTIs	Population-based observational study	NRTI exposure associated with 50% reduced risk of prediabetes and 27% reduced risk of progression from prediabetes to type 2 diabetes
NCT03179423 (Curtin et al, 2020)	Type 1 diabetes	Temelimab (anti-HERV-W Env monoclonal antibody)	Clinical trial (phase not specified)	No change in primary metabolic endpoints; exploratory: reduced hypoglycemic events at week 24, lower anti-insulin antibodies
**Aging**				
Anderson et al, 2026	Aging/biological age	NRTIs	Post hoc analysis	NRTI treatment associated with reduced epigenetic measures of biological aging and inflammation

This table summarizes studies that investigate the therapeutic benefit of targeting TE activity across five disease categories: cancer, neurological diseases, autoimmune diseases, metabolic disorders, and aging. Therapeutic strategies include nucleoside/non-nucleoside reverse transcriptase inhibitors (NRTIs/NNRTIs), which block the generation of TE-derived cytoplasmic DNA intermediates; monoclonal antibodies neutralizing TE-encoded proteins; and epigenetic modulators that reinforce TE silencing. For each study, the trial identifier or reference, disease condition, therapeutic strategy, study phase, and main outcome are reported.

*NRTI* nucleoside reverse transcriptase inhibitor, *NF-κB* nuclear factor kappa B, *IFN* interferon, *ISG* interferon-stimulated gene, *PSA* prostate-specific antigen.

### Cancer

By virtue of their capacity to mobilize and integrate into new genomic loci, TEs constitute an intrinsic source of mutagenesis. It is therefore not surprising that cancer was among the first disease contexts in which their uncontrolled activity was implicated (Nangia-Makker et al, 1998; Sciamanna et al, 2005; Burns, 2017; Payer and Burns, 2019). Genome-wide analyses indicate that derepression of evolutionarily young LINE-1 families is frequently associated with global hypomethylation, a hallmark of many tumors, suggesting that cancer-associated epigenetic dysregulation creates a permissive environment for TE activation (Burns, 2017; Rodriguez-Martin et al, 2020). Moreover, a pan-cancer analysis of 2954 cancer genomes identified 19,166 somatic LINE-1 insertions, with retrotransposition detected in ~35% of samples and particularly enriched in epithelial tumors such as liver, colorectal, and lung cancers; notably, a subset of these insertions targeted tumor suppressor genes, suggesting a potential contribution of LINE-1 mobilization to oncogenic transformation (Rodriguez-Martin et al, 2020). TE insertional events can target tumor suppressor genes or other cancer-relevant loci, thereby contributing to oncogenic transformation. In addition, TE mobilization can induce DNA double-strand breaks through endonuclease activity and promote genome instability via replication stress, structural rearrangements, and copy number alterations, even in the absence of successful integration (Gasior et al, 2006; Pizarro and Cristofari, 2016; Burns, 2017). Beyond direct mutagenic effects, TE-derived sequences can promote tumor progression by acting as regulatory elements that drive aberrant gene expression programs and confer a fitness advantage to cancer cells, a process referred to as onco-exaptation (Chuong et al, 2017; Jang et al, 2019). Many TEs harbor transcription factor binding sites that can function as alternative promoters or enhancers when epigenetic repression is relieved, thereby rewiring transcriptional networks linked to proliferation and cellular plasticity. This regulatory repurposing underscores the dual role of TEs as both sources of genomic instability and modulators of gene expression programs that support tumor progression.

Because LINE-1 mobilization requires reverse transcription of its RNA intermediate, pharmacological inhibition of endogenous reverse transcriptase (RT) represents one of the most direct approaches to limit TE activity in cancer. Several NRTIs, originally developed for the treatment of retroviral infections, have demonstrated anticancer activity in preclinical models. Early studies showed that inhibition of endogenous RT activity using lamivudine (3TC), zalcitabine, and stavudine reduced proliferation and promoted differentiation in transformed cells, supporting the hypothesis that cancer cells may rely on LINE-1 activity for maintenance of malignant phenotypes (Sciamanna et al, 2005; Carlini et al, 2010; Patnala et al, 2014; Chiou et al, 2021). In TP53-mutant colorectal cancer cells, treatment with 3TC reduced repeat element activity and impaired tumor cell migration, highlighting the contribution of endogenous retrotransposition to cancer-associated phenotypes (Rajurkar et al, 2022). Notably, the relationship between p53 and LINE-1 is more nuanced than simple suppression: recent work has shown that while p53 restricts LINE-1 retrotransposition, it can simultaneously activate LINE-1 transcription under genotoxic stress, a dual regulatory role with implications for the interpretation of TE activity in TP53-mutant tumors (Solovyov et al, 2025). Similarly, non-nucleoside RT inhibitors (NNRTIs), including nevirapine and efavirenz, have been shown to inhibit proliferation of multiple cancer cell types in vitro and in vivo, including prostate and pancreatic cancer and lung squamous cell carcinoma, where suppression of LINE-1 activity was associated with reduced tumor growth and altered metabolic pathways (Sciamanna et al, 2005; Hecht et al, 2015; Zhang et al, 2019).

These observations have motivated clinical repurposing of RT inhibitors in oncology. In a phase II clinical trial evaluating efavirenz in metastatic castration-resistant prostate cancer, treatment resulted in a subset of patients exhibiting stabilization of prostate-specific antigen levels, suggesting that inhibition of endogenous RT may influence disease progression (Houédé et al, 2014). Likewise, a phase II study investigating 3TC in TP53-mutant metastatic colorectal cancer reported disease stabilization in a fraction of patients (Parikh et al, 2020; Rajurkar et al, 2022). Ongoing efforts extend this approach to other malignancies, including high-grade serous ovarian cancer, where efavirenz is being evaluated in heavily pretreated patients (Lombard et al, 2025). A dose-escalation phase I trial further explored efavirenz across a broader range of solid tumors and non-Hodgkin lymphoma, establishing tolerability at doses up to 1800 mg and providing the pharmacological basis for subsequent disease-specific studies (NCT01878890). Although larger randomized studies are needed to determine therapeutic efficacy, these findings support the concept that endogenous RT activity represents a targetable vulnerability in at least a subset of tumors.

Complementary approaches have investigated whether suppression of TE-derived transcripts or proteins can directly impair tumor fitness. RNA interference targeting LINE-1 transcripts has been shown to reduce proliferation and tumorigenic potential in transformed cells, indicating that continued expression of LINE-1 contributes to maintenance of malignant phenotypes (Pizarro and Cristofari, 2016; Payer and Burns, 2019). Similarly, knockdown of HERV-K *Env* gene using shRNA significantly impaired proliferation, migration, and invasion in breast cancer models and reduced tumor growth and metastatic dissemination in pancreatic cancer xenograft models, supporting the functional relevance of TE-derived intermediates in cancer progression (Zhou et al, 2016; Li et al, 2017). These findings suggest that cancer cells may exhibit partial dependency on TE-derived transcripts or proteins, providing a rationale for therapeutic strategies aimed at directly suppressing TE expression. Epigenetic therapies have also been evaluated for their ability to modulate TE repression and stabilize genome integrity. In head and neck squamous cell carcinoma, soy isoflavone supplementation has been investigated for its ability to increase LINE-1 methylation levels, suggesting a potential chemopreventive effect associated with restoration of epigenetic stability (Rozek et al, 2019). Although these therapies are not exclusively designed to target TEs, modulation of LINE-1 methylation provides an accessible biomarker of global epigenetic regulation and may reflect therapeutic control of TE activity (Kosumi et al, 2020).

### Neurological diseases

Aberrant TE activity has been increasingly observed in both neurodegenerative and neurodevelopmental disorders, suggesting a contribution to disease pathophysiology. In the central nervous system (CNS), disruption of TE silencing can lead to their transcriptional activation in neuronal and glial cells, often associated with epigenetic alterations occurring during development, aging, or disease. For instance, TE expression has been shown to be higher in post-mortem brain biopsies of Alzheimer’s disease (AD) patients compared to non-AD controls, particularly in microglial cells (Guo et al, 2018; Roy et al, 2024; Kumar and Beck, 2026; Sun et al, 2018). In the developing mouse brain, loss of KRAB-TRIM28-mediated epigenetic repression leads to ERV re-expression and ERV-derived protein aggregate accumulation, ultimately resulting in microglial activation and inflammatory induction (Jönsson et al, 2021). Interestingly, TE dysregulation in AD has been proposed to be a consequence of pathogenic tau accumulation, which is responsible for both heterochromatin loss and piRNA depletion (Frost et al, 2014; Guo et al, 2018; Sun et al, 2018; Grundman et al, 2021; Ramirez et al, 2022). Moreover, c-Jun, upregulated in neurodegenerative diseases, promotes HERV-K expression through chromatin decondensation in AD neural progenitors and neurons (Scopa et al, 2023). Such TE dysregulation has been associated with neuroinflammation through the cytosolic accumulation of immunogenic dsRNA molecules (Ochoa et al, 2023) or DNA:RNA hybrids (Scopa et al, 2023), sensed by MDA5 and cGAS, respectively. Notably, 3TC treatment is sufficient to suppress pathological tau-induced neurotoxicity and locomotor deficits in a *Drosophila* tauopathy model (Sun et al, 2018), suggesting an important contribution of cGAS-STING cytosolic DNA sensing in AD pathogenesis.

Emerging clinical observations further support a functional link between TE activity and AD pathogenesis, while providing a rationale for therapeutic intervention. Recent retrospective analyses of large patient cohorts revealed that individuals exposed to NRTIs exhibited a significantly reduced incidence of AD compared to untreated HIV patients and non-HIV controls, suggesting that inhibition of endogenous RT activity may confer protection against disease onset (Chow et al, 2024; Magagnoli et al, 2025). It should be noted, however, that retrospective cohort studies of this type are subject to confounding: HIV patients receiving NRTIs differ from non-HIV controls in multiple ways, including comorbidities, co-medications, and healthcare access, and these associations should therefore be interpreted with caution pending prospective validation. Consistently, early interventional evidence indicates that pharmacological suppression of retrotransposon activity may be feasible and biologically relevant in patients. In a phase IIa pilot study, treatment of individuals with early AD using 3TC was well tolerated and associated with reductions in neuroinflammatory markers, alongside changes in amyloid-related biomarkers suggestive of decreased pathological burden (Sullivan et al, 2025). An ongoing phase II study is further assessing the effect of lamivudine on neurocognitive impairment biomarkers and IFN-stimulated gene expression in patients with mild cognitive impairment and positive AD biomarkers, with the IFN-I signature serving as a pharmacodynamic endpoint (NCT06519357). Although preliminary, these findings provide initial clinical support for a model in which TE-derived reverse transcription contributes to disease progression and highlight the therapeutic potential of targeting this pathway in AD. Complementary evidence from aged mice further supports this model: 3TC treatment improved cognitive function and reduced neuroinflammation in old mice, and TE transcripts were found to accumulate in human brain aging transcriptomes, suggesting that NRTI-mediated TE suppression may have broader relevance to age-related cognitive decline (Wahl et al, 2023). Nonetheless, these findings remain preliminary; the available clinical evidence consists largely of retrospective cohort analyses and a single small phase II trial, and definitive proof of therapeutic benefit in AD awaits adequately powered prospective studies.

Importantly, recent work provided direct support for a causal role of TE activation in neurodegeneration. In this work, targeted overexpression of LINE-1 in the mouse cerebellum was sufficient to induce Purkinje cell dysfunction, neurodegeneration, and ataxia, suggesting that LINE-1 activation alone may be sufficient to drive neuronal pathology in this model (Takahashi et al, 2022). LINE-1 upregulation was associated with DNA damage, gliosis, and disruption of calcium homeostasis, while treatment with 3TC mitigated disease progression (Takahashi et al, 2022). These findings provide the first direct in vivo evidence that TE derepression is not merely a downstream consequence of neurodegeneration but may act as an initiating pathogenic event.

A comparable link between TE dysregulation and neurodegenerative mechanisms has emerged in amyotrophic lateral sclerosis (ALS), where both LTR and non-LTR elements appear to contribute to neurodegeneration. In particular, HERV-K has been consistently implicated in ALS, with its expression detected in cortical and spinal neurons of affected individuals but not in healthy controls (Li et al, 2015), and its envelope protein measurable in neuronal extracellular vesicles from patient plasma, where levels correlate with disease severity and progression, supporting its potential as a non-invasive biomarker (Li et al, 2022). Consistently, HERV-K Env protein has also been detected in the cerebrospinal fluid of ALS patients and shown to exert direct neurotoxic effects (Steiner et al, 2022). Mechanistically, HERV-K expression is regulated by TDP-43, a central protein in ALS pathogenesis, whose nuclear depletion leads to widespread transcriptional dysregulation, including TE activation (Li et al, 2012). Functional studies demonstrated that neuronal expression of HERV-K or its envelope protein is sufficient to induce neurotoxic phenotypes, including neurite retraction, synaptic dysfunction, and progressive motor impairment in vivo (Li et al, 2015). More recent transcriptomic analyses across large patient cohorts have further refined this association, identifying specific HERV-K loci that are selectively upregulated in the spinal cord and cortex of ALS patients and correlate with disease-relevant gene expression signatures (Pasternack et al, 2024). In addition to direct neurotoxicity, HERV-K products can actively stimulate the immune system, as evidenced by their ability to trigger pro-inflammatory cytokine production, including IFN-γ, TNF-α, and IL-6, in CD19^+^/B cells from ALS patients (Arru et al, 2021).

Beyond HERVs, non-LTR elements such as LINE-1 also appear to play a role in ALS and related disorders. LINE-1 RNA accumulation has been linked to defects in RNA surveillance pathways, including impairment of the nuclear exosome targeting (NEXT) complex, resulting in aberrant stabilization of TE transcripts (Li et al, 2025). Notably, ASO-mediated LINE-1 knockdown mitigates chromosomal instability and improves neuronal survival in disease-relevant models, supporting a causal contribution of TE transcripts to neurodegeneration (Li et al, 2025). Consistent with a broader role for TE dysregulation in neuronal vulnerability, activation of HERV-K has also been shown to impair cortical neuron differentiation and function in human stem cell-derived cells and organoids, further highlighting the cell-type-specific and context-dependent effects of TE expression in the nervous system (Padmanabhan Nair et al, 2021).

Emerging clinical evidence further supports the therapeutic relevance of targeting TE activity in ALS. In a proof-of-concept study, a 24-week combination of the NRTIs abacavir and lamivudine, together with the integrase inhibitor dolutegravir (Triumeq), led to a significant reduction in HERV-K expression in the majority of treated patients, with TE levels rebounding upon treatment interruption (Gold et al, 2019; Garcia-Montojo et al, 2021). Notably, patients exhibiting a virological response showed a trend toward slower disease progression, as reflected by more modest declines in respiratory function and neuropsychological scores compared to non-responders. Building on this proof-of-concept evidence, the Lighthouse II study (NCT05193994) represented the most ambitious clinical test of this hypothesis to date: a global, randomized, double-blind, placebo-controlled phase III trial enrolling over 400 patients across 29 centers in Europe, Australia, and New Zealand, testing whether Triumeq could improve survival in ALS. However, a planned interim analysis conducted in April 2025 found no benefit on the primary endpoint of survival, leading to early termination of the trial. Although these results argue against a generalized survival benefit of HERV-K suppression in ALS, they do not exclude the possibility that a subset of patients with high HERV-K activity may respond, or that earlier intervention, alternative drug combinations, or patient stratification by HERV-K expression levels could yield different outcomes.

A more tentative extension of TE involvement beyond neurodegeneration concerns neurodevelopmental and psychiatric disorders, where evidence remains limited and largely correlative. In particular, increased expression of HERV-W has been reported in conditions such as schizophrenia, bipolar disorder, and autism spectrum disorders (Karlsson et al, 2001, 2004; Tamouza et al, 2021; Tovo et al, 2022; Rangel et al, 2024; Cipriani et al, 2025). Complementing these expression-based observations, recent genomic analyses have identified polymorphic non-reference transposable elements in the dorsolateral prefrontal cortex of individuals with schizophrenia, including loci that may act as cis-regulatory variants influencing gene expression and splicing, thereby providing a potential mechanistic link between TE insertional variation and disease risk (Modenini et al, 2023). Strengthening the genetic-epidemiological evidence, a transcriptome-wide association study (TWAS) in 792 post-mortem brain samples identified 26 HERV expression signals associated with psychiatric disorders, including five high-confidence risk loci with schizophrenia- and bipolar disorder-specific signals, providing the first evidence that specific HERV loci are genetically regulated in cis and contribute to psychiatric disease risk beyond expression associations alone (Duarte et al, 2024).

While human data were mainly associative, recent preclinical work provides initial mechanistic support: transgenic expression of the HERV-W Env protein in mice induces deficits in social memory, object recognition, and sensorimotor gating, alongside hippocampal transcriptomic alterations enriched for neurodevelopmental risk genes and synaptic pathways. These changes are accompanied by altered H3K4 methylation levels linked to increased LSD1 activity and can be partially rescued by pharmacological LSD1 inhibition (Herrero et al, 2025). These findings suggest that aberrant TE activation may contribute to neurodevelopmental phenotypes through epigenetic and synaptic mechanisms, although robust evidence in humans is still lacking.

### Autoimmune diseases

TEs have also been proposed as important contributors to the pathogenesis of autoimmune diseases, with increasing evidence of their dysregulation across multiple clinical contexts. In systemic lupus erythematosus (SLE) and related disorders such as primary Sjögren’s syndrome, several studies have demonstrated increased expression of retroelements, particularly LINE-1, in disease-relevant tissues (Kelly et al, 2018; Tokuyama et al, 2018; Mustelin et al, 2019; Kuriyama et al, 2021; Stearrett et al, 2021; Cutts et al, 2024). In a large-scale RNA-seq analysis, SLE patients were stratified into TE-high and TE-low subgroups, with elevated TE expression strongly correlating with activation of nucleic acid-sensing pathways and IFN-I signatures, and with neutrophils identified as a major source of TE-derived transcripts (Wang et al, 2024). Elevated levels of LINE-1 transcripts and proteins, including ORF1p/p40, have been detected in lupus nephritis kidneys and salivary gland biopsies, where they correlate with IFN-I expression and reduced DNA methylation at LINE-1 loci, indicating a loss of epigenetic repression (Mavragani et al, 2016, 2018). Recent data further support this model, linking distinct IFN response patterns to increased TE expression in leukocytes of SLE patients, thus reinforcing the association between TE activity and immune signaling (Ukadike et al, 2023; Arteaga-Vazquez et al, 2025). This model is further supported by experimental evidence demonstrating that disruption of DNA methylation directly promotes TE activation. Indeed, hypomethylation has been associated with the expression and mobilization of LINE-1 and HERV sequences in SLE patients (Nakkuntod et al, 2011; Fali et al, 2014). Consistently, pharmacological inhibition of DNA methyltransferases, using agents such as 5-azacytidine or procainamide, is sufficient to induce lupus-like disease in mice, establishing a functional link between epigenetic derepression, TE activation and SLE (Quddus et al, 1993). An ongoing clinical trial (NCT06356740) is evaluating the addition of the NRTIs abacavir and lamivudine to standard of care in patients with SLE. The study is designed to assess whether this combination can reduce the IFN-I signature over a 6-month treatment period.

Aicardi-Goutières syndrome (AGS) is an interferonopathy caused by mutations in genes involved in nucleic acid metabolism and sensing, including *TREX1*, *RNASEH2A*/*B*/*C*, *SAMHD1*, and *ADAR1*, which normally prevent the accumulation of endogenous nucleic acids (Rice et al, 2013; Crow and Manel, 2015). In particular, TREX1 has been shown to degrade cytosolic DNA generated during reverse transcription, and its deficiency results in the accumulation of LINE-, LTR-, and SINE-derived DNA intermediates capable of eliciting IFN-I responses (Stetson et al, 2008). Consistently, TREX1 deficiency leads to an increase in LINE-1-derived cDNA concentration and the development of a neuroinflammatory phenotype (Thomas et al, 2017). Additional studies have shown that other AGS-associated proteins, including SAMHD1 and ADAR1, also restrict retroelement immunogenicity by limiting reverse transcription or by editing dsRNA intermediates (Behrendt et al, 2013; De Reuver et al, 2021). In vivo, TREX1-deficient mice develop severe systemic inflammation and early lethality, consistent with uncontrolled activation of nucleic acid-sensing pathways (Stetson et al, 2008).

These observations provided the rationale for clinical studies exploring the efficacy of NRTI therapy in AGS patients. An initial open-label study reported that treatment with abacavir, lamivudine, and zidovudine reduced the IFN-I signature in AGS patients, although this effect was transient and rebounded after treatment cessation (Rice et al, 2018). Subsequent clinical evaluation in a small crossover trial involving 13 pediatric patients failed to demonstrate a sustained reduction in IFN-stimulated gene expression with either monotherapy or short-term combination therapy, despite a transient decrease observed at earlier time points, and was further limited by poor patient compliance as reported by the authors (Crow et al, 2025). These findings suggest that while RT inhibition can modulate IFN signaling, its effects may be limited in duration and influenced by treatment adherence and disease heterogeneity.

While the preceding examples illustrate the potential contribution of TE-derived nucleic acids to autoimmune pathology through innate immune sensing, multiple sclerosis (MS) provides a conceptually distinct example in which a TE-encoded protein may act as a primary pathogenic effector. Among TEs, the HERV-W family has been consistently implicated in MS pathogenesis. Increased HERV-W Env protein has been detected in the serum and within brain lesions of MS patients, with levels correlating with disease severity (Antony et al, 2004; Mameli et al, 2007; Perron et al, 2012). Importantly, this protein has been shown to exert direct pathogenic effects on oligodendrocyte precursors through TLR4 signaling, leading to the production of pro-inflammatory cytokines and amplification of inflammatory responses, ultimately resulting in a reduction in myelin protein expression (Kremer et al, 2013). Direct causal evidence for HERV-W Env pathogenicity in the CNS has been provided by a transgenic mouse model in which constitutive expression of HERV-W Env induced myelin repair deficits, polarization of microglia and astroglia toward neurotoxic states, and progressive axonal degeneration, thus recapitulating key features of MS neuropathology and establishing HERV-W Env as a sufficient driver of CNS damage in vivo (Gruchot et al, 2023). In addition to impairing remyelination, HERV-W Env has been implicated in axonal damage by promoting a neurodegenerative phenotype in microglial cells. Specifically, its expression in lesion-associated myeloid cells drives microglial polarization toward a state that enhances their interaction with myelinated axons, leading to structural axonal injury (Kremer et al, 2019).

To counteract the pathological activity of HERV-W Env, a monoclonal antibody, temelimab, has been developed to specifically neutralize this protein. In a phase II trial in relapsing-remitting MS patients, temelimab did not reduce acute inflammatory activity, but treatment at higher doses was associated with fewer new T1-hypointense lesions and showed trends toward reduced brain atrophy over time. These findings, together with a favorable safety profile, suggest a potential neuroprotective effect and support further evaluation of HERV-W Env neutralization, particularly in progressive disease stages (Kremer et al, 2019; Kornmann and Curtin, 2020; Hartung et al, 2022). In parallel, indirect strategies based on drug repurposing have also been explored. Notably, remission or improvement of MS symptoms has been reported in patients receiving highly active antiretroviral therapy (HAART), and although these effects are generally attributed to antiviral activity against exogenous infections such as HIV or Epstein-Barr virus, a contribution from inhibition of endogenous RT activity cannot be ruled out (Chalkley and Berger, 2014; Skarlis et al, 2017; Drosu et al, 2018).

Rheumatoid arthritis (RA) provides a further illustration of how TE dysregulation may contribute to autoimmune pathology. L1 elements are expressed in RA synovial tissue in association with global DNA hypomethylation, and epigenetic changes in synovial fibroblasts, including widespread hypomethylation at repetitive sequences, occur already at the earliest clinically apparent stages of disease, preceding the fully established destructive phenotype (Neidhart et al, 2000; Karouzakis et al, 2018). Consistent with a functional role for this derepression, retroelements MERVL and LINE-1 are markedly upregulated in RA joint tissues and osteoclasts, where they sustain cGAS-STING/NF-κB signaling and promote osteoclast differentiation and synoviocyte hyperproliferation; pharmacological suppression of reverse transcription with lamivudine attenuated this inflammatory cascade and reduced joint damage in a collagen-induced arthritis model, extending the therapeutic rationale for NRTI-based TE suppression to inflammatory arthritis (Shan et al, 2026).

### Metabolic disorders

Metabolism and epigenetic regulation are intrinsically interconnected, as cellular metabolic states directly influence the availability of key substrates and cofactors required for chromatin modifications (ElGendy et al, 2018; Jo et al, 2020). As a result, metabolic perturbations such as aging, dietary imbalance, and oxidative stress can drive widespread epigenetic remodeling (Wu et al, 2023). Within this framework, TEs, which are normally silenced by epigenetic mechanisms, represent particularly sensitive genomic regions that may become derepressed when epigenetic control is compromised.

Accumulating evidence suggests that TE dysregulation may play a role in the development of metabolic disorders, including obesity, type 2 diabetes, and metabolic dysfunction-associated steatotic liver disease (MASLD) (Kuehnen et al, 2012; Turcot et al, 2012; Lopes et al, 2019; Daouk et al, 2025; Youngson et al, 2025). Several studies have reported associations between altered methylation or expression of repetitive elements, particularly LINE-1 and Alu sequences, and metabolic traits or glycemic control, although results are not fully consistent across cohorts and tissues (Piyathilake et al, 2013; Martín-Núñez et al, 2014; Carraro et al, 2016; Thongsroy et al, 2017; Malipatil et al, 2018; Katsanou et al, 2023). Beyond serving as indicators of epigenetic disruption, activated TEs may exert functional effects on metabolic homeostasis through multiple mechanisms. These include the modulation of metabolic regulatory gene networks via TE-derived sequences (Kaur and Pociot, 2015), as well as the production of LINE- and SINE-derived IFN-inducing nucleic acids, thereby contributing to metabolic stress-associated chronic inflammation (Ambati et al, 2020; Daouk et al, 2025).

These insights have also opened the possibility of targeting TE activity as a therapeutic strategy in metabolic diseases. Pharmacological inhibition of RT activity has emerged as a promising approach. A large population-based study reported that exposure to NRTIs was associated with a significantly reduced incidence of diabetes, an effect supported by experimental evidence showing that 3TC improves insulin sensitivity and suppresses inflammasome activation in cellular and animal models. This effect appears to involve the reduction of TE-derived transcripts, particularly SINE RNAs, which can otherwise accumulate due to impaired catabolism and contribute to innate immune activation (Ambati et al, 2020). Extending this observation, a retrospective cohort study of more than thirty thousand HIV-positive veterans followed over 21 years found that NRTI exposure was associated with a 50% reduced risk of developing prediabetes and a 27% reduced risk of progression from prediabetes to overt type 2 diabetes, further supporting the therapeutic potential of RT inhibition in metabolic disease (Magagnoli et al, 2023). However, the therapeutic targeting of TE-related pathways is complex and may depend on the specific class of compounds and cellular context. Indeed, not all RT inhibitors exert beneficial effects. Certain NNRTIs, such as efavirenz and rilpivirine, can impair pancreatic β-cell function and viability through mechanisms involving oxidative stress, mitochondrial dysfunction, and endoplasmic reticulum stress (Maandi et al, 2022). In parallel, clinical studies have reported an increased susceptibility to bone fragility in patients receiving NRTI-based antiretroviral therapy (Brown and Qaqish, 2006; Grigsby et al, 2010). More recently, it was shown that NRTIs can directly impair osteocyte endocrine functions in vitro (Mangiavacchi et al, 2025). Collectively, these findings underscore the importance of carefully evaluating drug-specific toxicities and off-target effects when considering TE-targeting therapeutic strategies.

Beyond repurposed antiviral drugs, more direct approaches to modulate TE activity are beginning to emerge. Recent work by Daouk et al showed that dietary lipid stress can induce chromatin remodeling that derepresses LINE-1 elements in hepatocytes, triggering IFN-mediated inflammation. Importantly, targeted silencing of LINE-1 using ASOs restored chromatin homeostasis, reduced hepatic inflammation, improved insulin sensitivity, and attenuated fibrosis in a mouse model of MASLD (Daouk et al, 2025).

Whereas the preceding examples illustrate how TE-derived nucleic acids drive metabolic pathology through innate immune sensing, type 1 diabetes provides a parallel example, analogous to the HERV-W Env mechanism in MS, in which a TE-encoded protein is the primary pathogenic effector. Specifically, the pathological accumulation of HERV-W Env protein in the pancreas exerts cytotoxic effects on islet β- cells through TLR4 signaling, suggesting a potential role in the pathogenesis of type 1 diabetes (Levet et al, 2017; Tovo et al, 2020). In this context, the HERV-W Env-targeting monoclonal antibody temelimab, previously introduced as a therapeutic approach in MS, has also been evaluated in patients with type 1 diabetes. Despite no changes being observed in primary metabolic endpoints, exploratory analyses revealed a reduced frequency of hypoglycemic events at week 24 and lower levels of anti-insulin antibodies under treatment (Curtin et al, 2020).

### Aging

Aging is a systemic and multifactorial process characterized by progressive loss of cellular and organismal homeostasis, influenced by genetic, epigenetic, and environmental factors. Aging is also increasingly recognized as a dominant risk factor for a broad spectrum of chronic human diseases, including neurodegenerative disorders, metabolic dysfunction, and cancer. Despite their clinical heterogeneity, these conditions share a common biological denominator: a persistent, low-grade, sterile inflammatory state that gradually emerges and intensifies with age. This state, often referred to as “inflammaging”, reflects a chronic activation of innate immune pathways in the absence of overt infection and is now considered a central integrative feature linking aging to age-associated pathology.

In this scenario, a converging body of evidence suggests that TEs might represent a possible link between the aging process and the underlying chronic inflammation. Indeed, TE regulation deteriorates with age, as the age-associated epigenetic drift, including global DNA hypomethylation and heterochromatin erosion, weakens the silencing machinery that normally restrains TE activity (Wood et al, 2010; Chen et al, 2016). Consistently, elevated expression of repetitive elements has been observed across aging tissues and cellular senescence models (De Cecco et al, 2013; Wood et al, 2016). However, the extent to which increased TE expression translates into de novo transposition events in somatic cells remains debated. While some studies have reported age-associated retrotransposition events in model organisms, others have suggested that their frequency may be overestimated due to technical artifacts, indicating that somatic TE mobilization may occur at relatively low rates (Li et al, 2013; Evrony et al, 2016; Treiber and Waddell, 2017). These observations suggest that the contribution of TEs to aging may not primarily depend on the accumulation of insertional mutations. LINE-1 RNA itself, for instance, can drive heterochromatin erosion, independently of retrotransposition, by suppressing the histone methyltransferase SUV39H1, thereby reducing H3K9me3 and H3K27me3 marks and triggering senescent gene expression programs; depletion of LINE-1 RNA with an ASO in fibroblasts from patients with progeroid syndromes restored heterochromatin marks, reversed epigenetic age, and extended lifespan in a progeria mouse model, identifying LINE-1 RNA as a direct therapeutic target in premature aging syndromes (Della Valle et al, 2022).

Moreover, increasing evidence indicates that TE activation contributes to aging through the generation of immunostimulatory nucleic acids. Indeed, LINE-1 derepression in aged cells and mice can also lead to cytoplasmic cDNA accumulation that activates cGAS-STING signaling, driving an IFN-I response that can be prevented by 3TC (De Cecco et al, 2019; Simon et al, 2019). Moreover, in the context of age-related macular degeneration, LINE-1-mediated reverse transcription of Alu RNAs produces cytoplasmic Alu cDNA intermediates that accumulate in retinal pigment epithelium (RPE) cells, ultimately leading to IFN production, inflammasome activation and RPE cell death (Fukuda et al, 2021a, 2021b). This can be inhibited by treatment with high doses of efavirenz (Fukuda et al, 2021a).

This TE-associated activation of IFN signaling has been proposed to contribute to the reinforcement of a late-stage senescence-associated secretory phenotype (SASP), which is widely considered a hallmark of aging (Coppé et al, 2010; De Cecco et al, 2019). Importantly, the magnitude, duration, and composition of the SASP also exert a profound influence on tissue repair dynamics: while transient SASP can support tissue repair and immune-mediated clearance of damaged cells, its persistent activation can drive fibroblast activation, extracellular matrix deposition, and impaired tissue regeneration, ultimately contributing to the development of fibrosis (Demaria et al, 2014; Hiebert et al, 2018; Baechle et al, 2023). Thus, TE-induced inflammatory pathways may not only reinforce cellular senescence but also participate in the transition from chronic inflammation to fibrotic tissue remodeling, further connecting TE dysregulation to major age-related dysfunctions.

The fibrotic conditions described below illustrate how the TE-driven innate immune activation and SASP reinforcement outlined above manifest in specific age-associated pathological contexts, each converging on the same upstream molecular events (TE derepression, cytoplasmic nucleic acid accumulation, and innate immune sensor engagement) as drivers of tissue-specific fibroinflammatory remodeling. In idiopathic pulmonary fibrosis (IPF), a prototypical age-associated fibrotic disease, oxidative stress has been hypothesized to activate TEs, potentially altering gene expression programs and amplifying inflammatory responses in lung tissue (Rostami and Bradic, 2021). Consistently, transcriptional profiling of lung cells from IPF patients revealed altered LINE-1 expression patterns compared to healthy controls, suggesting that TE activation may contribute to the inflammatory and fibrotic remodeling of the alveolar compartment (Rostami and Bradic, 2021). Similarly, in chronic kidney disease, increased ERV expression driven by epigenetic derepression has been linked to activation of cytosolic nucleic acid sensors, including RIG-I and MDA5, which detect cytosolic dsRNA structures formed by ERV transcripts, and cGAS-STING, which senses the cytoplasmic cDNA intermediates produced by ERV reverse transcription, the same molecular events driving inflammaging and SASP reinforcement described above, ultimately promoting cytokine production and immune cell infiltration (Dhillon et al, 2023). Notably, genetic or pharmacological inhibition of these sensing pathways, as well as suppression of reverse transcription with 3TC, was sufficient to attenuate renal fibroinflammation, providing direct functional evidence for a causal role of TE-derived nucleic acids in fibrotic pathology (Dhillon et al, 2023). A comparable therapeutic approach has been demonstrated in cardiac aging: LINE-1 ASOs delivered via extracellular vesicles attenuated cardiac senescence, reduced cGAS-STING activation, alleviated cardiac fibrosis and improved cardiac function after myocardial infarction in mouse models, extending the ASO-based TE suppression strategy to an age-related cardiovascular context (Fu et al, 2025). Furthermore, LINE-1 was found to be upregulated in calcified arteries of patients with chronic kidney disease, where LINE-1-derived cDNA directly activates cGAS-STING in vascular smooth muscle cells, promoting osteogenic reprogramming and vascular calcification; NRTI treatment suppressed this pathway and attenuated calcification in two mouse models, and a cross-sectional study of 1785 patients confirmed that NRTI use is independently associated with a lower prevalence and severity of vascular calcification (Ma et al, 2025).

Taken together, the evidence across these diverse pathologies converges on a common principle: in each setting, derepression of transposable elements generates immunostimulatory nucleic acids and, in some cases, protein products that feed chronic inflammation, genome instability, and tissue dysfunction, making TE activity a mechanistically grounded and potentially unifying therapeutic target. Yet the clinical translation of this concept remains at an early stage. Most associations rest on observational or preclinical data; direct measures of TE activity in patients are still scarce, and no interventional trial has yet demonstrated unequivocal clinical benefit of a TE-targeting strategy. Realizing this potential will require more selective agents, biomarkers that stratify patients by TE activity, and controlled trials designed to test whether modulating TE activity alters disease course.

Because TE activity rises progressively with age as epigenetic silencing erodes, age acts as a systemic modifier and potential confounder across essentially all the conditions considered here, most of which are themselves age-associated. Two consequences follow. First, the baseline level of TE expression against which a disease-specific signal must be distinguished is not fixed but shifts with the age of the individual, complicating both the identification of pathogenic TE activity and its use as a biomarker. Second, and more consequentially for therapy, a treatment that suppresses TE activity may act not only on the disease-driving elements but also on the diffuse, age-related component of TE derepression, whose physiological or even adaptive roles remain poorly defined. This raises the possibility that the therapeutic window, the appropriate degree of suppression, and the balance of benefit and risk are all age-dependent, and that interventions validated in one age group or disease stage may not translate directly to another. Disentangling disease-specific from age-associated TE activity, therefore, emerges as a central challenge for the rational deployment of TE-modulating therapies.

## Harnessing transposable element activation for therapeutic benefit

In contrast to strategies aimed at suppressing TE activity, an alternative and conceptually complementary approach seeks to therapeutically harness their controlled reactivation. This approach can be broadly framed as a set of modalities that exploit the intrinsic immunogenicity of TEs to elicit a controlled inflammatory response. In cancer, this concept has been primarily explored through the induction of an antiviral response in tumor cells, enhancing their immune recognition. Beyond oncology, transient TE activation may also engage physiological inflammatory programs that support tissue repair and regeneration. An important caveat applies to all reactivation-based strategies: deliberate induction of TE transcriptional activity carries the theoretical risk of promoting de novo retrotransposition events, with potential consequences including insertional mutagenesis and genomic instability. The long-term genomic safety of sustained or repeated epigenetic TE reactivation remains poorly characterized and warrants careful evaluation in preclinical models before clinical translation.

### Transposable element reactivation as an immunotherapeutic strategy in cancer

The recognition that certain epigenetic anticancer therapies can induce TE expression and trigger antiviral-like immune responses in cancer cells marked a turning point in the field, providing an unexpected link between chromatin regulation and tumor immunogenicity. These early observations revealed that pharmacological disruption of epigenetic silencing in cancer cells is sufficient to elicit a “viral mimicry” state, characterized by the activation of IFN pathways downstream of endogenous nucleic acid sensing, thereby increasing the visibility of cancer cells to the immune system (Leonova et al, 2013; Chiappinelli et al, 2015; Roulois et al, 2015; Jang et al, 2024; Larsen et al, 2026). More recently, this paradigm has broadened even further, as a growing number of conventional and targeted anticancer therapies, including chemotherapeutic agents, radiotherapy, and kinase inhibitors, have been shown to converge on similar immunostimulatory outcomes through TE reactivation. Ionizing radiation, CDK4/6 inhibitors, EZH2 inhibitors, and MEK inhibitors have each been shown to induce TE reactivation and trigger downstream antiviral immune responses in cancer cells, mirroring the effects originally described for epigenetic drugs (Goel et al, 2017; Deblois et al, 2020; Morel et al, 2021; Du et al, 2023; Cortesi et al, 2024). Paradoxically, drug-resistant tumor states can reinforce epigenetic TE silencing to evade viral mimicry, a vulnerability that can be targeted by combining the resistance-inducing agent with an epigenetic drug to restore TE expression and immune recognition (Deblois et al, 2020).

The identity of the TE subfamilies and innate sensing pathways engaged varies with the epigenetic perturbation and tumor context. Early studies with DNA methyltransferase inhibitors (DNMTis) showed that TE-derived dsRNA can activate intracellular RNA-sensing pathways and induce robust IFN responses in ovarian, colorectal, renal, and other cancer models (Chiappinelli et al, 2015; Roulois et al, 2015; de Cubas et al, 2020). Among the most immunogenic elements identified are ERVs and inverted-repeat Alu elements, which represent a major source of dsRNA following epigenetic derepression (Mehdipour et al, 2020). A recent study showed that the inhibition of the m6A methyltransferase METTL3 in colorectal cancer increases the accumulation of Alu- and other RE-derived dsRNAs, identifying RNA methylation as an additional layer regulating the immunogenic potential of TE-derived transcripts and the magnitude of viral mimicry responses (Wang et al, 2026). Importantly, combining epigenetic agents can further amplify TE reactivation and immune signaling. For example, combined DNMT and histone deacetylase (HDAC) inhibition induces stronger TE derepression than either treatment alone and promotes the expression of cryptic TE-derived transcripts and putative neoantigens (Brocks et al, 2017; Goyal et al, 2023). In parallel, several other enzymes have emerged as key modulators of viral mimicry competence. Factors such as ADAR1, SETDB1, SETD2, and LSD1 normally restrict the immunogenic potential of TE-derived nucleic acids, and their inhibition enhances IFN signaling, immune cell infiltration, and TE-derived antigen presentation (Griffin et al, 2021; Gomez et al, 2022; Burbage et al, 2023; Li et al, 2023; Merlotti et al, 2023; Sheng et al, 2018). Collectively, these findings support the idea that multiple epigenetic and post-transcriptional pathways converge to regulate the threshold for TE-mediated immune activation in cancer cells.

These mechanistic insights have motivated clinical trials combining epigenetic priming with checkpoint inhibitors. A randomized phase II trial of azacitidine, a DNMTi previously shown to activate TEs (Ohtani et al, 2020), combined with pembrolizumab in patients with chemotherapy-refractory metastatic colorectal cancer demonstrated that epigenetic priming increased immune cell infiltration and upregulated interferon-stimulated gene signatures consistent with TE-driven innate immune activation, with disease control achieved in a subset of patients (Kuang et al, 2022). Similarly, a phase II trial of guadecitabine combined with pembrolizumab in platinum-resistant ovarian cancer reported on-treatment induction of ERV transcripts and IFN pathway activation in tumor biopsies, supporting a viral mimicry mechanism of immune sensitization (Landon et al, 2026). A phase II neoadjuvant window trial of decitabine followed by pembrolizumab in HER2-negative breast cancer demonstrated increased stromal tumor-infiltrating lymphocytes and PD-L1 expression on-treatment, consistent with viral mimicry-mediated immune priming, and achieved a pathological complete response rate of 40% in triple-negative breast cancer patients (Bear et al, 2025). Finally, a phase I clinical trial evaluating the EZH2 inhibitor tazemetostat in combination with pembrolizumab in patients with anti-PD-1-resistant head and neck squamous cell carcinoma successfully established the recommended phase II dose, while patient enrollment and efficacy evaluation for the expansion phase are currently ongoing (Oppelt et al, 2025). Taken together, these data validate the viral mimicry framework as a tractable therapeutic strategy, while underscoring the need for patient selection based on baseline TE expression and immune microenvironment status to identify contexts where epigenetic priming most effectively converts immune exclusion into immune engagement.

A conceptually distinct but complementary strategy seeks to harness TE-derived peptides as tumor antigens, shifting the therapeutic logic from innate immune activation to the direct engagement of adaptive cytotoxic immunity. Indeed, IFN signaling induced under viral mimicry conditions drives the upregulation of antigen-processing machinery and MHC-I, thereby increasing the capacity of tumor cells to present peptides to cytotoxic T cells (Luo et al, 2018). Importantly, TE reactivation itself contributes to this process by generating neoantigens, as TE-derived peptides can be presented on MHC-I, expanding the repertoire of targets available for immune recognition and, in some cases, providing shared tumor-associated antigens across patients (Saini et al, 2020).

Among TE families, LTR elements, particularly HERVs, have proven to be an especially productive source of tumor-specific antigens. The first demonstration that HERV-derived peptides can be directly recognized by cytotoxic T cells came from studies in renal cell carcinoma, where HERV-E and HERV-R envelope sequences were found to be presented on MHC-I and to stimulate tumor-reactive CD8⁺ T cells (Takahashi et al, 2008; Cherkasova et al, 2016; Smith et al, 2018). The therapeutic potential of this observation has since been explored in other tumor types: in melanoma, CAR-T cells engineered to target HERV-K envelope peptides exerted substantial antitumor activity in preclinical models (Krishnamurthy et al, 2015). In parallel, computational immunopeptidomic pipelines have enabled the systematic identification of HERV-derived epitopes capable of priming de novo CD8⁺ T cell responses and engaging pre-existing tumor-infiltrating lymphocytes in breast cancer (Bonaventura et al, 2022). More recently, Maldonado et al developed a therapeutic vaccine targeting the ERV envelope protein ERVMER34-1, highly expressed across human carcinomas but largely absent from normal adult tissues (Maldonado et al, 2025). This approach effectively controlled established tumors in preclinical models and, when combined with checkpoint blockade or an IL-15 superagonist, further expanded both ERVMER34-1-specific and endogenous neoepitope-reactive T cells, suggesting that shared TE-derived antigens can act as focal points to amplify broader antitumor immunity (Maldonado et al, 2025). The most advanced clinical translation of this concept to date is an ongoing phase I trial evaluating autologous T cells engineered to express a T cell receptor specific for a HERV-E-derived peptide presented on HLA-A*11:01 in patients with metastatic clear cell renal cell carcinoma (ccRCC). The rationale rests on the tumor-selective reactivation of HERV-E in ccRCC, which renders this antigen effectively tumor-specific and shared across patients. The trial, sponsored by the NIH National Heart, Lung, and Blood Institute, enrolled 17 patients and has completed accrual, representing the first-in-human demonstration that a HERV-derived antigen can be therapeutically targeted with engineered T cells (Barisic et al, 2024). Clinical trials exploiting TE-derived antigens and TE-driven immune responses in cancer are summarized in Table 2.

**Table 2:** Clinical trials targeting transposable element-derived antigens and TE-driven immune responses in cancer.

Trial/reference	Condition	Therapeutic strategy	Phase	Main outcome
**Cancer**				
NCT02260440 (Kuang et al, 2022)	Chemotherapy-refractory metastatic colorectal cancer	Pembrolizumab + azacitidine	Phase II	Well tolerated; on-treatment demethylation correlated with CD8 + TIL infiltration and TE upregulation.
NCT02900560 (Landon et al, 2026)	Platinum-resistant ovarian cancer	Pembrolizumab + guadecitabine	Phase II	On-therapy IFN signaling and CD8 + T-cell density upregulated; ERV-associated signatures enriched in responders.
NCT02957968 (Bear et al, 2025)	HER2-negative breast cancer	Pembrolizumab + decitabine	Phase II	Well tolerated; reduced M-MDSCs and increased TIL and PD-L1 expression, sensitizing tumors to subsequent chemotherapy.
NCT04624113 (Oppelt et al, 2025)	Recurrent or metastatic head and neck squamous cell carcinoma	Pembrolizumab + tazemetostat	Phase I	RP2D: tazemetostat 800 mg BID; safe and tolerable
NCT03354390	Metastatic clear cell renal cell carcinoma	HERV-E TCR-engineered T cells	Phase I	Ongoing; primary endpoint: safety by day 21; secondary endpoints: overall response rate, progression-free survival and overall survival.

This table summarizes clinical trials that exploit the immunogenic properties of TEs in oncology. Two complementary approaches are represented: epigenetic priming, in which epigenetic modulators are combined with immune checkpoint blockade to induce a viral mimicry state in tumor cells, enhancing their immune recognition; TE-derived antigen-directed cellular therapies, including T cells engineered to express a T cell receptor specific for a HERV-derived tumor antigen. For each study, the trial identifier, reference, disease condition, therapeutic strategy, study phase, and main outcome are reported.

*DNMTi* DNA methyltransferase inhibitor, *RP2D* recommended phase II dose, *TCR T* cell receptor, *TIL* tumor-infiltrating lymphocytes, *PD-L1* programmed death-ligand.

Antigenicity is unlikely to be restricted to canonical HERV proteins. TE-derived promoters and splice sites can generate chimeric TE-gene transcripts encoding novel open reading frames whose products are entirely absent from normal tissues, effectively expanding the repertoire of cancer-specific antigens beyond conventional mutational neoepitopes. Proteogenomic analyses across 33 tumor types identified multiple TE-driven chimeric proteins presented on MHC-I molecules and detectable at the tumor cell surface, highlighting their accessibility to both T cell- and antibody-based therapies (Shah et al, 2023). These findings raise the possibility that the repetitive genome may represent a largely untapped reservoir of shared tumor antigens, potentially overcoming one of the central limitations of neoantigen-based immunotherapy, namely the high degree of patient-specific heterogeneity. Notably, Shah et al further proposed that a therapeutic vaccine targeting only 20 recurrent TE-chimeric proteins could theoretically achieve coverage of more than 75% of tumors across 27 cancer types, suggesting that TE-derived antigens may provide the basis for broadly applicable “off-the-shelf” cancer immunotherapies (Shah et al, 2023).

### Beyond pathology: transposable elements as regulators of tissue regeneration

The early phase of tissue regeneration is typically characterized by a transient inflammatory response that is necessary for effective repair (Choi et al, 2023). During this stage, the combination of inflammatory signaling, chromatin remodeling and rapid cell proliferation may create a permissive environment for TE derepression (Angileri et al, 2022). Consistent with this idea, increased TE expression has been reported in regenerating tissues across phylogenetically distant species, including axolotls, sea cucumbers, brittle stars and earthworms (Mashanov et al, 2012; Zhu et al, 2012; Mashanov et al, 2020; Shao et al, 2020). Whether TE reactivation is a cause or consequence of the regenerative process remains unclear; however, the evolutionary conservation of this association has led to the hypothesis that it may represent more than a by-product of epigenetic relaxation (Angileri et al, 2022). TE promoters are extensively bound by pluripotency- and lineage-specific transcription factors, indicating that TEs participate in the establishment of developmental transcriptional programs (Ohnuki et al, 2014; Wang et al, 2014; Ito et al, 2017). Moreover, LINE-1 transcripts can participate in chromatin organization through interactions with KAP1 and Nucleolin in embryonic stem cells, thereby contributing to the regulation of developmental potency (Percharde et al, 2018). It has therefore been proposed that regeneration may partially rely on the transient reactivation of these TE-associated developmental networks (Angileri et al, 2022). Supporting this possibility, studies in the hematopoietic system have shown that stress-induced chromatin remodeling leads to the production of TE-derived transcripts that may contribute to the inflammatory signaling required for stem cell exit from quiescence and regenerative activation (Lefkopoulos et al, 2020; Clapes et al, 2021).

Growing evidence suggests that cellular senescence may have context-dependent reparative functions when transiently induced. In zebrafish, senescent cells emerge transiently during fin regeneration, and their elimination impairs tissue repair, while analogous transient senescent populations have been described in mouse models of skeletal muscle, skin, liver and lung injury, where they appear to support regeneration through SASP-mediated paracrine signaling (Demaria et al, 2014; Da Silva-Álvarez et al, 2020; Young et al, 2022). More recently, evidence from skin injury suggests that a very early, rapid senescence-like response may also occur at wound edges, preceding the classical senescence program and contributing to efficient wound closure. Importantly, this early response appears to be temporally restricted, being required for proper repair initiation but dispensable at later stages (Valdivieso et al, 2026). Since TE derepression is both a driver and a consequence of senescence, and since TE-derived nucleic acids can promote SASP-related inflammatory pathways through cGAS-STING and type I IFN signaling, the temporal regulation of TE activity may be tightly linked to the controlled inflammatory dynamics required for tissue repair (Tunbak et al, 2020). While transient activation of nucleic acid-sensing pathways may contribute to regenerative signaling, persistent stimulation can instead sustain chronic inflammation, fibrosis and tissue degeneration. In a rat model of cerebral ischemia, for example, cytosolic LINE-1 cDNA accumulates shortly after injury and is associated with prolonged cGAS-STING activation, infarct expansion and neurodegeneration, whereas NRTI-mediated suppression of reverse transcription attenuates these effects and improves cortical recovery (Gamdzyk et al, 2020). Similarly, chronic cGAS-STING activation has been implicated in pulmonary and kidney fibrosis, where pharmacological inhibition of STING reduces myofibroblast activation and extracellular matrix deposition (Chung et al, 2019; Ou et al, 2023). Conversely, acute activation of the same pathway has been associated with beneficial regenerative outcomes in models of peripheral nerve injury and irradiation-induced intestinal damage (Fischer et al, 2017; Wang et al, 2023). Together, these findings are consistent with the idea that the regenerative or pathological consequences of TE-associated inflammatory signaling may depend on its magnitude and temporal persistence, although the mechanistic basis of this distinction remains to be established in direct experimental models.

Initial evidence supporting the existence of a transient post-injury TE wave in mammalian tissue was recently provided by Mangiavacchi et al, who reported that bone fracture induces an early and transient upregulation of repetitive elements coinciding with the inflammatory phase of repair (Mangiavacchi et al, 2024). Notably, TE expression in human bone was also found to correlate with local mechanical stress and bone mineral density, suggesting a possible link between TE activation and tissue remodeling dynamics. Functionally, TE-derived dsRNA sensing in osteoblasts promoted mineralized matrix deposition and altered the osteoblast secretory program, generating paracrine signals capable of propagating pro-repair responses to neighboring cells (Mangiavacchi et al, 2024). Importantly, this pathway appears to be specifically driven by L1 RNA rather than L1 reverse-transcribed DNA intermediates or canonical cGAS-STING signaling, as pharmacological inhibition of reverse transcriptase activity or cGAS did not impair L1 RNA-induced mineralization. In contrast, L1 RNA engages PKR, leading to phosphorylation of eIF2α and downstream modulation of translational and inflammatory programs. Consistently, inhibition of PKR attenuated both inflammatory gene activation and matrix mineralization, supporting a model in which L1 RNA-PKR signaling acts as a key effector pathway linking TE activity to osteogenic responses during fracture repair. While further studies across additional tissues and independent replication are needed to establish the generality of these findings, it is tempting to speculate that regulated, transient TE activation may also occur in mammalian tissues, where it could contribute to the orchestration of multicellular regenerative processes.

From a therapeutic perspective, innate immune pathways engaged by TE-derived nucleic acids are increasingly being investigated in regenerative settings, even though no clinical interventions currently target TEs directly. In this context, the synthetic dsRNA analog poly(I:C), which mimics endogenous dsRNA species generated during tissue injury, has been the most widely used experimental tool. By activating TLR3, poly(I:C) recapitulates an early wound-associated innate immune signal, and its exogenous administration significantly improved re-epithelialization, granulation tissue formation, and neovascularization in murine skin wound models, and was also reported to accelerate wound closure in a small clinical study in patients undergoing laser plastic surgery (Lin et al, 2012; Nelson et al, 2015). At the cellular level, poly(I:C) drives keratinocyte collective migration through autocrine IL-8 secretion and bFGF upregulation, accompanied by a partial epithelial-to-mesenchymal transition that facilitates sheet migration without compromising barrier identity (Takada et al, 2017). These effects are partly mediated by TLR3-dependent induction of retinoic acid production and IL-6/STAT3 activation, thereby promoting hair follicle neogenesis and epidermal regeneration (Nelson et al, 2015; Kim et al, 2019). Beyond the skin, TLR3 activation by poly(I:C) has been shown to confer immunosuppressive properties on periodontal ligament stem cells, inducing IDO, HLA-G expression, and Treg expansion through endosomal TLR3 signaling, suggesting that the same damage-sensing axis can prime mesenchymal progenitors across tissue types toward a pro-regenerative, immunomodulatory phenotype (Chaikeawkaew et al, 2020).

A particularly promising application of this principle is the pharmacological priming of mesenchymal stem cells (MSCs) with poly(I:C) prior to therapeutic use. MSCs are multipotent stromal cells with well-established immunomodulatory and tissue-reparative properties, currently under clinical evaluation for a range of inflammatory and degenerative conditions. However, their therapeutic potency is variable, and strategies to enhance their paracrine output before administration have attracted considerable interest (Trigo et al, 2025; Sandonà et al, 2021). TLR3 stimulation with poly(I:C) reprograms MSCs toward an immunosuppressive phenotype characterized by upregulation of anti-inflammatory mediators, antiviral proteins, and a remodeled extracellular vesicle cargo enriched in innate immune defense proteins (Pierce and Kurata, 2021). Interestingly, ex vivo exposure of blood from severe COVID-19 patients to poly(I:C)-primed MSCs recalibrated dysregulated myeloid responses toward a pro-resolving phenotype, demonstrating activity in a clinically relevant human system (Souza-Moreira et al, 2022). These findings establish poly(I:C) priming as a tractable strategy to enhance MSC therapeutic function. However, whether the beneficial effects induced by poly(I:C) mechanistically overlap with the physiological responses triggered by endogenous TE-derived nucleic acids during tissue repair remains to be elucidated.

Modulation of cGAS-STING signaling represents a parallel avenue for regenerative intervention. Direct evidence that transient cGAS-STING activation is part of the normal repair program comes from skeletal muscle: following barium chloride-induced injury in mice, cGAS, STING, and the transcriptional co-regulator YY1 are coordinately upregulated at day 3 and return to baseline by day 7 as myogenic differentiation proceeds, with YY1 physically interacting with STING to modulate the early inflammatory response (Yan et al, 2025). The pro-regenerative potential of deliberate STING agonism has been demonstrated across multiple tissue types. In the skin, topical application of cGAMP accelerated wound healing in mice through enhanced IFN-β production, promoting immune cell recruitment to the wound site (Mizutani et al, 2020). In the nervous system, activation of cGAS-STING signaling has been linked to regenerative responses in both central and peripheral neurons. In the CNS, direct neuronal stimulation with cGAMP promotes axon regeneration after optic nerve injury; in the peripheral nervous system, injury-induced induction of IFN-γ upregulates cGAS in Schwann cells, leading to cGAMP production that acts as an immunotransmitter to support spontaneous axon regrowth, collectively suggesting that cGAS-STING signaling can function as a cell-autonomous repair pathway in neural tissues (Wang et al, 2023). Importantly, STING agonists have now entered phase I/II oncology trials in humans, including ADU-S100, MK-1454, and IMSA101, establishing the safety and pharmacokinetic profile of systemic innate immune activation via this pathway (Meric-Bernstam et al, 2022; Harrington et al, 2025; Jacoby et al, 2025). Although these trials target tumor immunity rather than tissue repair, they provide a translational scaffold demonstrating that controlled, transient STING activation is clinically feasible in humans. Together, these findings suggest that harnessing the innate immune pathways naturally activated by endogenous TE-derived nucleic acids may represent a tractable therapeutic strategy for tissue repair, with the key challenge being the controlled delivery of transient, spatially restricted stimulation.

## Conclusions and future perspectives

The body of evidence reviewed here converges on a unifying principle: transposable elements are not merely genomic fossils but emerging, important actors in the molecular dialog between the genome, the environment and the immune system. However, the extent to which these associations reflect causal mechanisms remains an open question in most disease contexts. Indeed, their derepression generates immunostimulatory nucleic acids, including double-stranded RNAs and cytoplasmic cDNA intermediates, which engage pattern-recognition receptors such as cGAS, MDA5, RIG-I, and TLR3, ultimately converging on type I IFN signaling. Yet the molecular details underlying this shared axis differ substantially across contexts. In cancer, ERV-derived dsRNAs and LINE-1 cDNA engage MDA5/RIG-I and cGAS-STING, respectively, with the balance varying by tumor type and only a subset of ERV subfamilies driving robust innate activation in any given context (Cañadas et al, 2018; Chen et al, 2021; Ohtani et al, 2020). In neurodegeneration, HERV-K RNA-DNA hybrids activate cGAS-STING in neurons while tau-induced TE-derived dsRNAs engage dsRNA sensors in glia, and LINE-1 upregulation shifts microglial state through a distinct transcriptional program (Scopa et al, 2023; Roy et al, 2024; Ochoa et al, 2023). In autoimmune disease, LINE-1-derived cDNA engages cGAS-STING while LINE-1 RNA can activate both RIG-I and MDA5, depending on cellular context, with type-I IFNs themselves upregulating LINE-1 in myeloid cells and creating a cell-type-restricted feed-forward loop (Zhao et al, 2018; Mavragani et al, 2016; Le et al, 2025). In senescent cells, LINE-1 cDNA and ERV-derived dsRNA activate cGAS-STING and MDA5-MAVS in parallel, two TE families engaging distinct sensors within the same cell (De Cecco et al, 2019; Di Giorgio et al, 2024). This mechanistic diversity means that TE activity cannot be treated as a single uniform therapeutic target, but rather as a family of related yet context-specific vulnerabilities, each requiring tailored intervention strategies.

Beyond direct innate immune sensing, TE-derived regulatory elements can contribute to the transcriptional control of IFN-stimulated genes, while TE-encoded peptides presented on MHC-I molecules expand the repertoire of antigens available for immune surveillance. The biological consequences of this activity are highly context-dependent. When transient and tightly controlled, TE-induced innate immune signaling may support physiological processes, such as activation of stem cells and other cell types (Lefkopoulos et al, 2020; Clapes et al, 2021). Conversely, when persistent or unresolved, the same pathways can sustain chronic inflammation and promote disease. This duality emerges across the pathophysiological contexts discussed in this review, encompassing the oncogenic and neurotoxic effects of sustained LINE-1 and HERV activity in cancer and neurodegeneration, the type I IFN-driven inflammatory cascades observed in SLE, AGS, MS, and metabolic stress, and the feed-forward loop connecting epigenetic erosion, TE derepression, and age-related dysfunction. Emerging observations from in vivo and in vitro regenerative models further suggest that transient TE activation may also participate in tissue repair responses (Angileri et al, 2022; Mangiavacchi et al, 2024), although current evidence remains limited and the mechanistic contribution of TEs to regeneration is still partially understood.

These observations have given rise to two complementary therapeutic strategies. One aims to suppress pathological TE activity, thereby limiting the chronic generation of immunostimulatory nucleic acids through approaches such as RT inhibition, sequence-specific ASO targeting, or reinforcement of epigenetic silencing. The other seeks to harness controlled TE reactivation, exploiting the immunogenic properties of TE-derived nucleic acids and peptides to enhance tumor immune recognition and potentiate cell-based therapies. Although conceptually distinct, both approaches ultimately converge on the same molecular axis: the sensing of TE-derived nucleic acids and the downstream activation of IFN signaling. Therapeutic interventions targeting this pathway are now being explored across a growing spectrum of diseases, ranging from cancer and autoimmune disorders to age-related conditions. Nevertheless, despite the compelling mechanistic rationale, clinical translation remains at an early stage. For most indications reviewed here, the clinical evidence remains limited to retrospective studies, small early-phase trials, or trials with negative or inconclusive outcomes. Realizing the therapeutic potential of TE biology will require more selective tools, improved patient stratification, robust biomarkers of TE activity, and a deeper understanding of the cell-type- and tissue-specific mechanisms that govern TE activity.

Among the suppressive strategies, nucleoside RT inhibitors represent the most clinically advanced approach, benefiting from decades of safety data accumulated in the treatment of HIV and hepatitis B infection. Epidemiological studies and early interventional trials have provided encouraging signals across AD, ALS, AGS, SLE, and metabolic disorders, and recent data suggest that NRTI exposure may improve some hallmarks of aging. Moreover, their oral bioavailability and established pharmacokinetic profiles facilitate rapid clinical repurposing. It should be noted, however, that NRTIs are not selective for TE-encoded reverse transcriptases and also inhibit other polymerases, including mitochondrial DNA polymerase γ and telomerase, and exert anti-inflammatory effects through NLRP3 inflammasome inhibition independently of RT activity (Holec et al, 2017). Additional limitations include mitochondrial toxicity, hepatic steatosis, myopathy, and peripheral neuropathy, particularly with older compounds (Holec et al, 2017). NRTIs have also been associated with nephrotoxicity and adverse skeletal effects, including reduced bone mineral density and impaired osteocyte function (Mangiavacchi et al, 2025; Holec et al, 2017); these pleiotropic mechanisms complicate the attribution of any observed clinical benefit specifically to TE suppression. Newer compounds such as tenofovir alafenamide carry substantially lower toxicity profiles while retaining pharmacological activity (Sax et al, 2015). Moreover, RT inhibitor effects may be transient, as illustrated by the IFN-I rebound observed in AGS patients after treatment cessation (Rice et al, 2013; Crow et al, 2025). The negative outcome of the Lighthouse II Phase III trial in ALS illustrates that a strong mechanistic rationale does not necessarily translate into clinical benefit, highlighting the importance of biomarker-guided patient stratification and the development of more selective TE-targeting therapies.

Antisense oligonucleotides offer a complementary and potentially more precise suppressive strategy, enabling sequence-specific knockdown of individual TE transcripts or subfamilies. Proof-of-concept studies have demonstrated their efficacy across diverse disease-relevant contexts: LINE-1 ASOs mitigated chromosomal instability and improved neuronal survival in ALS models (Li et al, 2025), reduced cardiac senescence and fibrosis after myocardial infarction (Fu et al, 2025), reversed epigenetic age in progeria models (Della Valle et al, 2022), and restored chromatin homeostasis and attenuated hepatic inflammation in MASLD (Daouk et al, 2025). The high sequence specificity of ASOs allows targeting of individual TE families or even specific loci, and the durable knockdown achievable with cerebrospinal fluid-delivered ASOs, as demonstrated for approved drugs targeting SMA and ALS, establishes a regulatory and clinical precedent for this modality in neurological disease (Mercuri et al, 2018; Sumner and Miller, 2024). A notable feature of TE-targeting ASOs is that the repetitive nature of transposons may allow a single oligonucleotide to silence different targets simultaneously, thereby amplifying therapeutic impact. However, this same sequence homology across TE subfamilies also raises the risk of unintended off-target effects, including collateral silencing of non-target transcripts.

The principal limitation of ASO-based therapies remains delivery: crossing the blood–brain barrier to target the CNS requires intrathecal administration or conjugation to receptor-targeting vehicles, and the phosphorothioate backbone can elicit immunostimulatory and hepatotoxic effects (Amanat et al, 2022; Ramasamy et al, 2022). In addition, manufacturing costs remain substantial due to the multistep chemical synthesis, purification requirements, and extensive backbone and sugar modifications needed for clinical-grade oligonucleotides. Advances in manufacturing technologies and conjugation chemistry, including antibody-ASO conjugates, cholesterol-conjugated heteroduplex oligonucleotides, and lipid nanoparticle formulations, are rapidly improving tissue-specific bioavailability and may bring systemic delivery of TE-targeting ASOs within clinical reach.

Epigenetic modulators offer a bidirectional therapeutic tool: they can either reinforce TE silencing, by restoring repressive chromatin states disrupted in disease, or reactivate TEs to induce viral mimicry and enhance tumor immunogenicity. DNMT and HDAC inhibitors are already approved for hematological malignancies and are being evaluated in combination with immune checkpoint inhibitors across multiple solid tumor types, with clinical trials in colorectal, ovarian, and breast cancer demonstrating on-treatment induction of TE-derived dsRNA, IFN pathway activation, and increased immune infiltration consistent with viral mimicry (Kuang et al, 2022; Bear et al, 2025; Landon et al, 2026). EZH2 and LSD1 inhibitors extend this principle to additional chromatin regulatory axes, and their combination with checkpoint blockade is under active clinical investigation (Oppelt et al, 2025; Sheng et al, 2018). The key advantages of this strategy are the availability of approved agents with established clinical profiles, and the capacity to simultaneously reactivate TE-derived antigens, upregulate antigen-processing machinery, and sensitize tumors to immune recognition through a single intervention. The central limitation is the lack of locus specificity: genome-wide demethylation or hyperacetylation carries the risk of activating oncogenic programs alongside TE reactivation, and the high inter- and intra-tumor heterogeneity of epigenetic alterations complicates patient selection (Suraweera et al, 2025; Scandolara et al, 2026). Emerging locus-specific epigenetic editing platforms, including dCas9 fused to DNMT or KRAB repressor domains, hold the promise of targeted TE silencing or activation at defined genomic loci, potentially overcoming the specificity limitations of current pharmacological agents (Gilbert et al, 2013; Liu et al, 2016; Stepper et al, 2017).

The identification of TE-derived peptides as tumor antigens opens a conceptually distinct therapeutic avenue: the development of anticancer vaccines targeting HERV and TE-chimeric epitopes. A key potential advantage of this approach is that, unlike conventional neoantigen vaccines, which are strictly patient-specific, subsets of TE-derived antigens may be shared across patients with the same tumor type by virtue of their common epigenetic derepression, raising the prospect of broadly applicable immunotherapies, although the extent and consistency of such sharing across patients and tumor types remain to be systematically established. Computational proteogenomic analyses have identified recurrent TE-chimeric proteins presented on MHC-I across multiple tumor types, and preclinical studies targeting HERV-derived antigens with engineered T cells and therapeutic vaccines have demonstrated antitumor activity, with the first-in-human trial of HERV-E-specific TCR-engineered T cells in renal cell carcinoma providing initial clinical proof-of-concept (Shah et al, 2023; Barisic et al, 2024; Maldonado et al, 2025). Modern mRNA vaccine platforms, validated by the clinical success of personalized neoantigen vaccines in melanoma (Weber et al, 2024), provide a scalable and rapidly manufacturable delivery vehicle for TE-derived antigen sequences, and their combination with epigenetic priming agents could further amplify antigen expression and T cell priming. The principal challenges are the risk of central tolerance to TE-derived neoantigens, potential immune evasion through MHC-I downregulation, and the limited clinical validation to date, as most evidence remains preclinical (Buonaguro and Tagliamonte, 2020). Nonetheless, the convergence of improved antigen discovery pipelines, mRNA delivery technologies, and the clinical infrastructure established by neoantigen vaccine trials positions TE-derived antigen vaccines as one of the most promising near-term translational opportunities in this field. Realizing this potential will require prospective clinical trials with integrated biomarker programs to define the patient populations, tumor types, and combination partners in which TE-targeted immunotherapy is most likely to succeed.

It should be emphasized that the therapeutic principles discussed here apply almost exclusively to retrotransposons, the transposable element classes with established roles in innate immune sensing and the most direct relevance across the disease contexts reviewed. DNA transposons, although valuable as engineering tools for ex vivo cell therapies such as CAR-T manufacturing, have not to date been targeted as endogenous therapeutic substrates in any clinical trial, and for this reason fall outside the scope considered here. Finally, it might be tempting to speculate that the transient TE induction observed after tissue injury is not merely a tolerated side effect of chromatin relaxation, but a functionally integrated component of the acute inflammatory program required for efficient repair. Consistent with this idea, the inflammatory response to injury is not a tissue-wide event but is spatially and cellularly compartmentalized: specialized tissue-resident “sentinel” cells are poised to sense local damage signals and rapidly initiate a localized, calibrated inflammatory response (Antonelli and Kushner, 2017; Peiseler and Kubes, 2018; Reyes et al, 2022). Whether TE-driven innate immune signaling operates preferentially within such spatially restricted sentinel populations remains speculative. Similarly, while it is conceivable that dysregulated TE activity in aged tissues contributes to impaired regeneration, given that TE expression is broadly deregulated with age and that the pro- to anti-inflammatory transition is delayed, as illustrated in skeletal muscle (Walter et al, 2024), this connection has not been directly demonstrated. More broadly, Feschotte and colleagues have proposed that the ability to mount and subsequently repress a transient TE wave after injury may represent a conserved checkpoint for regenerative competence (Angileri et al, 2022), a concept originally formulated in highly regenerative organisms and not yet directly tested in mammals.

A final caveat that applies across all disease contexts reviewed here concerns the technical challenges of detecting and quantifying retrotransposon activity in vivo. The repetitive nature of TE sequences creates substantial mapping ambiguity in short-read sequencing data, and most studies to date have operated at the family or subfamily level, which obscures locus-specific heterogeneity and may lead to overestimation or misattribution of TE-derived signals. These limitations have been extensively discussed elsewhere (Lanciano and Cristofari, 2020), and developing robust locus-level quantification frameworks will be essential to reliably interpret the biological and clinical significance of TE dysregulation across disease contexts. A further layer of complexity arises from the relationship between TE transcription and downstream immunological outcomes. Because TE transcripts are partitioned between the nucleus and cytoplasm, their abundance does not necessarily translate into protein expression, and neither RNA nor protein levels reliably predict antigen presentation or immunogenicity (Noronha et al, 2024). Most studies measure TE activity at the RNA level, yet the functional outputs relevant to therapy (cytoplasmic nucleic acid accumulation, innate immune sensor engagement, and peptide presentation on MHC-I) each depend on additional regulatory steps that remain poorly characterized in disease contexts.

Taken together, the work reviewed here makes clear that TEs are not passive genomic relics but active molecular participants whose dysregulation underlies a surprisingly wide spectrum of human disease, and whose controlled modulation, whether suppressive or activating, opens therapeutic avenues whose exploration has only just begun.

### Pending issues

- Clarifying epistatic relationships and possible druggable steps impacting the causal contribution of TE activity to acute and chronic inflammatory responses, and determining to what extent TEs act as primary drivers of inflammation or as passive passengers that amplify pre-existing inflammatory signals.
- Dissecting the transcriptional and epigenetic mechanisms governing individual TE families and identifying the cell populations in which specific TE subfamilies become dysregulated across distinct pathophysiological contexts.
- Disentangling the disease-specific component of TE activity from the diffuse, age-associated derepression that accompanies normal aging, and determining how this distinction should inform the timing, degree, and patient selection of TE-modulating therapies across age-related conditions.
- Identifying predictive biomarkers and stratification frameworks to guide the selection of patients most likely to respond to TE-targeting therapies across diverse disease settings.
- Developing TE-targeting strategies with enhanced specificity for individual TE families or genomic loci, evaluating whether such selectivity is sufficient to minimize off-target effects on physiological TE functions and host gene regulation.
- Defining the physiological role of transient TE activation in tissue repair and its potential contribution, via innate immune signaling, to the coordination of inflammatory, proliferative, and remodeling phases of regeneration.

## Supplementary information

Peer Review File

## **Glossary**

**Alu/SINE**: Short, very abundant transposable elements that cannot retrotranspose on their own but hijack the LINE-1 machinery to copy themselves.
**Antisense oligonucleotide (ASO)**: A short synthetic nucleic acid designed to bind and silence a specific RNA; a way to target individual TE transcripts.
**CAR-T (chimeric antigen receptor T cell)**: A patient’s T cells genetically engineered to recognize a chosen target antigen; explored here to direct immune killing against cells displaying TE-derived antigens.
**cGAS-STING**: A cellular sensor that detects cytosolic DNA (including TE-derived DNA) and triggers the interferon response.
**HERV (human endogenous retrovirus)**: Relics of ancient retroviral infections (~8% of the genome); no longer able to replicate but still able to produce RNA and proteins.
**Inflammaging**: The chronic, low-grade inflammation that accumulates with age in the absence of infection.
**Interferon (IFN) response**: A set of antiviral signals released by cells; chronic, inappropriate activation drives inflammation in several TE-associated diseases.
**LINE-1 (L1)**: The only human transposable element family still able to move autonomously (~20% of the genome); a major source of TE activity in disease.
**MHC-I (major histocompatibility complex I)**: The system that displays protein fragments on the cell surface so T cells can inspect them and detect abnormal or foreign proteins.
**Neoantigen**: A novel protein fragment that the immune system can recognize as foreign; exploitable in cancer immunotherapy.
**NRTIs (nucleoside reverse transcriptase inhibitors)**: Nucleoside analogs originally developed to block HIV reverse transcriptase; now being repurposed to suppress TE activity.
**Retrotransposition**: The full “copy-and-paste” process by which a retrotransposon inserts a new DNA copy of itself into the genome.
**Retrotransposon**: A transposable element that copies itself via an RNA intermediate that is converted back into DNA and inserted elsewhere in the genome.
**RIG-I/MDA5**: Cellular sensors that detect double-stranded RNA (including TE-derived RNAs) and trigger antiviral signaling.
**Senescence**: A state of permanent cell-cycle arrest; senescent cells secrete an inflammatory mix (the senescence-associated secretory phenotype) linked to aging.
**Viral mimicry**: A state in which TE-derived RNA or DNA is sensed by the cell as if it were a virus, triggering an antiviral immune response.
