# Supplementary material for: The therapeutic potential of transposable element activity modulation
Source: EMBO Mol Med. 2026 Oct 3;18(11):4200–26. doi: 10.1038/s44321-026-00528-z (PMC13748579; doi:10.1038/s44321-026-00528-z)
Supplement: Supplementary file 1 [file 44321_2026_528_MOESM1_ESM.pdf]

EMBO Molecular Medicine

The therapeutic potential of transposable element activity modulation

Gabriele Morelli, Marco D'Onghia, Arianna Mangiavacchi, and Valerio Orlando

Corresponding author: Valerio Orlando (valerio.orlando@kaust.edu.sa)

Review Timeline:

Submission Date:	8th Jun 26
Editorial Decision:	20th Jul 26
Revision Received:	6th Aug 26
Accepted:	14th Aug 26

Editor: Zeljko Durdevic

Transaction Report:

(Note: With the exception of the correction of typographical or spelling errors that could be a source of ambiguity, letters and reports are not edited. Depending on transfer agreements, referee reports obtained elsewhere may or may not be included in this compilation. Referee reports are anonymous unless the Referee chooses to sign their reports.)

20th Jul 2026

Dear Prof. Orlando,

Thank you for the submission of your manuscript to EMBO Molecular Medicine. We have now received feedback from the three reviewers who agreed to evaluate your manuscript. As you will see from the reports below, the referees are positive about its interest and timeliness, however, they also raise serious criticisms that should be fully addressed in a major revision of the current manuscript. Particular attention should be given to implementing referee #1 suggestions incl. shortening the manuscript and adding discussion on DNA transposons as therapeutic tools.

I would also like to ask you to amend the following:
1) Please add corresponding author's email address to the title page.
2) Please remove figure from the manuscript and leave their legends on the end of the file. Upon editorial assessment we may send the article to our graphic artist who will re-draw the figures for style and clarity. Therefore, please submit your revised figures as individual high-resolution files in an editable format (e.g. PDF with the option 'editable labels' checked).
3) Add callouts for Figure 2 and Tables 1 and 2.
4) Please provide Tables in an editable format and in grayscale (e.g. normal .doc tables) and place them together with their legends after Figure legends.
5) Please add "Glossary". The glossary is meant to explain some of the terms used for laymen. Could you please identify terms that may need an "explanation"?
6) Add "Disclosure and competing interests statement". We updated our journal's competing interests policy in January 2022 and request authors to consider both actual and perceived competing interests. Please review the policy https://www.embopress.org/competing-interests and update your competing interests if necessary.
7) Add "Acknowledgments" section that should include information of all sources of funding.
8) Correct the reference citation in the reference list. Where there are more than 10 authors on a paper, 10 will be listed, followed by "et al.". Please remove DOIs for published articles. Please check "Author Guidelines" for more information. https://www.embopress.org/page/journal/17574684/authorguide#referencesformat
9) Please specify author contributions in our submission system. CRediT has replaced the traditional author contributions section because it offers a systematic machine-readable author contributions format that allows for more effective research assessment. You are encouraged to use the free text boxes beneath each contributing author's name to add specific details on the author's contribution. More information is available in our guide to authors: https://www.embopress.org/page/journal/17574684/authorguide#authorshipguidelines
10) As part of the EMBO Publications transparent editorial process initiative EMBO Molecular Medicine will publish online a Review Process File (RPF) to accompany accepted manuscripts. This file will be published in conjunction with your paper and will include the anonymous referee reports, your point-by-point response and all pertinent correspondence relating to the manuscript. Let us know whether you agree with the publication of the RPF.
11) Please provide a point-by-point letter INCLUDING my comments as well as the reviewer's reports and your detailed responses (as Word file).

I hope that the referees' comments do not prove too problematic to address and I look forward to reading your next version.

Yours sincerely,

Zeljko Durdevic

Zeljko Durdevic
Senior Editor
EMBO Molecular Medicine

*** IMPORTANT INFORMATION ***

When submitting your revised manuscript, please include:

1) a .doc formatted version of the manuscript text (including Figure legends and tables)
2) Separate figure files

3) a letter INCLUDING the reviewer's reports and your detailed responses to their comments.

Also, and to save some time should your paper be accepted, please read below for additional information regarding some features of our research articles:

1) Glossary: EMBO Molecular Medicine articles will be accompanied by a glossary explaining some of the terms used for laymen. I identified the following:

____, ____, ____

Could you please help us in identifying terms that may need an "explanation" other terms that we can add to the glossary.

2) For more information: This is a short list of related web links for further consultation by the readers. Could you identify some relevant ones? Examples are patient associations, OMIM related links, databases, authors websites, etc.

3) Pending issues: At the end of each article we will have a box highlighting issues that still need further studies and where research efforts should converge (we call this the Pending issues box). From my reading I would say:

----

but I can see there may be many more. Could you work on this as well?

4) Disclosure and competing interest statement: Please include a statement declaring any competing commercial interests in relation to your submitted work.

5) Please note that we now mandate that all corresponding authors list an ORCID digital identifier. This takes <90 seconds to complete. We encourage all authors to supply an ORCID identifier, which will be linked to their name for unambiguous name identification.

Currently, our records indicate that the ORCID for your account is 0000-0002-4906-8511.

Please click the link below to modify this ORCID:
Link Not Available
-

Thank you,

Zeljko Durdevic

Zeljko Durdevic
Editor
EMBO Molecular Medicine

***** Reviewer's comments *****

Referee #1 (Remarks for Author):

This is an ambitious and timely review with a clear central concept: transposable elements (TEs) should be viewed as therapeutic targets that can either be suppressed or intentionally activated depending on disease context. That is an attractive organizing principle and distinguishes it from existing reviews.
The review has a excellent conceptual organization. It is organized around therapeutic strategies rather than TE classes, which makes it easy to follow. The parallel between suppressing TE activity and exploiting TE activation is elegant and provides a coherent framework.
The review covers an impressive range of topics, such as cancer, neurodegeneration, autoimmune disease, metabolism, aging and regeneration. It integrates multiple therapeutic strategies, including NRTIs, ASOs, epigenetic drugs, viral mimicry, vaccines , and CAR/TCR therapy.
The figures and tables are informative. Figure 1 summarizes innate immune sensing pathways well and Figure 2 provides a useful overview of therapeutic strategies. The tables summarizing clinical trials are also valuable.
The manuscript is generally well written. However it is too long. At ~75 pages with ~1800 references lines it reads almost like a

book chapter.

Overall, I think the manuscript has the potential to become an influential review because of its therapeutic perspective, but it currently leans too heavily toward advocacy. A more balanced treatment of causality, clinical evidence, and mechanistic uncertainty would significantly improve its scientific rigor.

My recommendation: Major revision

Major concerns

1. The review consistently overstates the maturity of the field. Throughout the manuscript, the distinction between association and causality, biological plausibility and clinical efficacy, and hypothesis and established mechanism becomes blurred. This advocacy-driven tone weakens the otherwise excellent review. My main concern is that the review in its current form is overly advocacy-driven, sometimes blurring the distinction between established biology, plausible hypotheses, and genuine therapeutic evidence.

o The review occasionally overstates causality, particularly in disease areas where the evidence remains largely associative. Throughout the review the language frequently shifts from "TE activation is associated with ..." to "TE activation drives disease". For many diseases the evidence simply does not support such strong wording. For example, there is a little direct evidence that TE activation is the initiating pathogenic event in Alzheimer's disease, schizophrenia, metabolic syndrome, aging. Instead, we have correlation and conclusions from using animal models. The authors should be more disciplined throughout. The review would be substantially stronger if the authors adopted a more balanced and critical tone.

o Given its therapeutic emphasis, the review would benefit from a more critical appraisal of the available clinical evidence. In fact, the clinical evidence is much weaker than the review implies. For many therapeutic approaches the evidence consists of retrospective studies, clinical trial (phase I and or trials with negative outcomes). For example, the authors appropriately mention that Lighthouse II failed (ALS). However, their narrative still suggests that TE inhibition remains a promising therapeutic avenue. The conclusion should be rewritten to emphasize that biological plausibility is not equal to clinical efficacy. Likewise, AD, SLE aging or metabolism are all discussed much more optimistically than current evidence justifies.

2. One thing that stood out to me is that the authors cite their own work extensively, particularly in the regeneration section and in the discussion of bone biology. While this is understandable because they have contributed to this area, some of these sections read more like promotion of an emerging hypothesis than an objective review. They should make clearer where independent replication is still lacking.

3. A surprisingly large fraction of the review revolves around reverse transcriptase inhibitors (NRTIs) (e.g. lamivudine). This gives an impression that TE therapeutics largely means repurposing HIV drugs. The readers may incorrectly conclude that NRTIs specifically validate TE biology. The authors should mention that NRTIs might have mitochondrial toxicity, inflammasome inhibition, and unrelated to LINE-1 RT. These issues deserve considerably deeper discussion.

4. It would be important to distinguish 'transposition' from 'transcriptional activity' much more carefully. Throughout the review, these concepts are sometimes conflated. In many disease contexts, the pathogenic evidence concerns TE-derived RNAs, dsRNAs, or cDNAs and innate immune sensing, not bona fide insertional retrotransposition. Less attention is given to the consequences of insertional retrotransposition, which might result in genomic instability, insertional mutagenesis, that could accompany prolonged TE activation. That distinction is fundamental and deserves stronger emphasis, particularly in a review intended to guide therapeutic development.

5. TE biology is sometimes oversimplified, and several sections compress a great deal of mechanistic complexity. For example: TE-derived inflammation is presented almost as TE activation, followed by dsRNA/cDNA activating signalling, ultimately contributing to disease. Importantly however, in reality different TE families in different cell types sense different sensing pathways and different chromatin states might produce very different outcomes. The review would benefit from more nuance.

6. The regeneration section is interesting, however it becomes speculative. It shifts from evidence into hypothesis without clear boundaries. For example, transient TE activation coordinates regeneration is presented almost as an emerging paradigm. At present this is largely speculative outside a handful of systems. The authors should more clearly distinguish known from proposed.

7. The title refers broadly to "transposable elements", yet the review almost exclusively discusses retrotransposons. DNA transposons are only briefly introduced and subsequently disappear. At minimum, the authors should explain why they intentionally restrict their discussion to retrotransposons, or modify the title accordingly. Alternatively, they could briefly discuss DNA transposons as therapeutic tools (e.g. Sleeping Beauty or piggyBac for CAR-T engineering), which would broaden the translational perspective.

8. A therapeutic review should discuss also discuss TE identifiaction and quantification problems (e.g. mapping ambiguity, family-level vs locus-level analyses, locus-specific quantification, etc.) Without this, readers may underestimate how difficult it is to determine whether TEs are truly activated.

9. The authors mention Barbara McClintock and regulatory functions. But there is little discussion of host-TE coevolution/exaptation, and why therapeutic manipulation might have unintended consequences on co-opted TEs.

10. The authors might put TE activation into a larger context. The The authors almost equate TE RNA with activation of the innate immunity. Yet the field increasingly appreciates that innate immune sensors respond to many forms of endogenous aberrant RNA, incuding cryptic transcription, intron retention, read-through transcription, defective RNA processing, aborted transcripts. See for example, the 'Unwanted transcript hypothesis': doi: 10.1038/s41576-023-00686-7. Placing TE-derived RNAs into this broader landscape would make the review much more balanced.

11. Many disease sections repeat the same structure: TE activation resulting in IFN activation, NRTIs, clinical trial ending by "more work needed". The review could be shortened by 20-30%.

Overall, I think the manuscript has the potential to become an influential review because of its therapeutic perspective, but it

currently too long and leans too heavily toward advocacy. A more balanced treatment of causality, clinical evidence, and mechanistic uncertainty would significantly improve its scientific rigor.

Referee #2 (Remarks for Author):

The review is well-written and timely. The authors did a great job reviewing current suppression of TE therapies in clinical trials. In particular, the notion that rapidly advancing yet incomplete understanding of how NRTIs could be used as anti-transposon therapies is very relevant. The authors nicely describe the anti-cancer therapies based on transposons and categorizing them, as well identify the positive aspects of each. "Pending Issues" section communicated truly relevant areas of research that TE-studying labs all around the world are working on. The review makes a unique contribution to the field by creating a framework where TE could also be used as therapeutic agents.

Areas of improvement:

• Some figures appear with low resolution.
• Introduction of HERV-type TE in paragraph on Neurological diseases (line 196+) is well-integrated within this specific paragraph but lacks mentioning anywhere else before in text, especially in the introduction.
• It would be helpful to the reader to show the typical LINE-1 and HERV structures in the introduction to allow the reader for better orientation later in the text?
• Additionally, it would be helpful to elaborate on different cytosolyc nucleic acid sensors mentioned (line 543)? What activates each of them, and how is it relevant to aging mentioned earlier in paragraph?
• In line 24, could the authors elaborate on the "diseases of our time", to connect their introduction to future discussions of AD, ALS, cancer, etc?

Referee #3 (Remarks for Author):

Interesting review that organizes and summarizes very well current literature on the relationship between TEs and immune pathways. The introduction was concise and well written and provided good motivation for the piece. The reference base is current, with a substantial fraction of 2024-2026 primary literature.

I appreciated the description of the therapeutic suppression of TEs across human diseases, and particularly the summary of the recent clinical trials of RT inhibitors in cancer. Similarly, the description of the contribution of TEs transcripts to neurological disorders and how they can be targeted was also very rich and comprehensive. The link between TEs and autoimmune diseases is also described in detail with, once again, an emphasis on some of the relevant clinical trials. This includes a detailed description of what is known about the contributions of TEs to MS and RA. I also appreciated the description of the contributions of TEs to metabolomic disorders, which complemented very effectively the other sections.
The links between TE expression and aging are also relevant and well described in the following section. The description is well balanced, quite informative and focuses on the likely TE-driven activation of IFN signaling. Once again, this section included a presentation of some of the relevant human studies.

After this broad overview of how TEs can potentially be suppressed to treat human diseases, the article moved into a description of how TEs could be overexpressed for therapeutic benefits. The description of immune activation in cancer mediated by TEs is well presented and includes the role of TE-derived transcripts, products and also peptides (both canonical and chimeric), which can act as tumor antigens. This potential complementary therapeutic approach is well known in cancer ("viral mimicry") but is also well described for other conditions. I didn't know about the potential role of TE activitation in regeneration and found that section interesting.

Finally, the conclusion does a great job at summarizing the piece and adding some complementary information on what was described in the main text. The piece ends with several important pending issues. I thought Fig 1 and 2 were well done.

Comments:
Page 32-33 I found the section describing the role of TE activation in regeneration interesting but perhaps a bit long compared to some of the other sections.

I wonder if you could expand a bit in the discussion about the implications of TEs in aging and how it potentially impacts how they could be used as therapeutic modulators. I think this is confounder that probably impacts all other conditions if TEs are to be used as therapeutic modulators. I personally would have included something about this in the pending issues as well.

There is very little discussion on the challenges in measuring and characterizing TE expression (e.g. multi-mapping ambiguity, read-through / pervasive transcription as a confound, antisense vs sense, full-length vs fragment, translational gap: transcription ≠ translation ≠ presentation ≠ immunogenicity). Perhaps adding a section somewhere on some of the potential limitations of some of the existing studies stemming from this fact?

Only Figure 1 was presented in the main text, I didn't see the references to Fig 2, Table 1-2.

The authors addressed the remaining editorial issues.

14th Aug 2026

Dear Prof. Orlando,

We are pleased to inform you that your manuscript is accepted for publication and is now being sent to our publisher to be included in the next available issue of EMBO Molecular Medicine, pending the final figure adjustments by our graphic designer, who will contact you for your approval of the figures.

Your manuscript will be processed for publication by EMBO Press. It will be copy edited and you will receive page proofs prior to publication. Please note that you will be contacted by Springer Nature Author Services to complete licensing information.

Should you be planning a Press Release on your article, please notify the production team via this form in order to coordinate publication and release dates: https://forms.cloud.microsoft/e/eH4A8JVP6g

If you have any questions, please do not hesitate to contact the Editorial Office. Thank you for your contribution to EMBO Molecular Medicine.

Yours sincerely,
Zeljko Durdevic

Zeljko Durdevic
Senior Editor
EMBO Molecular Medicine
